# Oxidative Susceptibility of Coumarin–1,2,3-Triazole–Isatin Hybrids: Integrating In Silico CYP-Mediated Metabolism Prediction with Electrochemistry–Mass Spectrometry

**DOI:** 10.3390/ph19101568

**Published:** 2026-10-01

**Authors:** Aleksandar Dimkovski, Darija Krzhovska, Vladimir Dobričić, Milena R. Simić, Evgenija Mihajloska, Aleksandra Kapedanovska Nestorovska, Aleksandra Grozdanova, Ana Poceva Panovska, Zoran Sterjev

**Affiliations:** 1 Institute for Pharmaceutical Chemistry, Faculty of Pharmacy, Ss Cyril and Methodius University in Skopje, Majka Tereza 47, 1000 Skopje, North Macedonia; darija@ff.ukim.edu.mk (D.K.); emihajloska@ff.ukim.edu.mk (E.M.); alka@ff.ukim.edu.mk (A.K.N.); agrozdanova@ff.ukim.edu.mk (A.G.); zost@ff.ukim.edu.mk (Z.S.); 2 Department of Pharmaceutical Chemistry, Faculty of Pharmacy, University of Belgrade, Vojvode Stepe 450, 11221 Belgrade, Serbia; vladimir.dobricic@pharmacy.bg.ac.rs; 3 Department of Organic Chemistry, Faculty of Pharmacy, University of Belgrade, Vojvode Stepe 450, 11221 Belgrade, Serbia; milena.simic@pharmacy.bg.ac.rs; 4 Institute for Applied Chemistry and Pharmaceutical Analysis, Faculty of Pharmacy, Ss Cyril and Methodius University in Skopje, Majka Tereza 47, 1000 Skopje, North Macedonia; anpo@ff.ukim.edu.mk

**Keywords:** electrochemistry–mass spectrometry, electrochemical oxidation, cytochrome P450, in silico metabolism prediction, oxidative metabolism, coumarin–1,2,3-triazole–isatin hybrids

## Abstract

**Background/Objectives**: Oxidative metabolism can influence the development of bioactive compounds by affecting metabolic stability and cytochrome P450 (CYP) interactions. This study aimed to assess the CYP-related properties and oxidative susceptibility of 21 coumarin–1,2,3-triazole–isatin hybrids by integrating in silico prediction with online electrochemistry–mass spectrometry (EC–MS). **Methods:** All 21 derivatives were evaluated in silico for CYP substrate and inhibitor profiles and CYP-mediated transformations. Four structurally representative derivatives (**6a1**, **6b1**, **6c1**, and **6c2**) were selected for analysis by directly coupled online electrochemistry–mass spectrometry (EC–MS). Compounds **6a1**, **6b1**, and **6c1** formed a C2–C4 homologous series, whereas **6c2** was the 5-fluoro analogue of **6c1**. **Results:** CYP3A4 was the isoform most consistently associated with substrate classification, while CYP3A4 inhibition probabilities remained close to the classification threshold. GLORY identified aromatic hydroxylation as the predominant top-ranked transformation across the series. Linker length had little influence on the principal predicted oxidation sites. EC–MS revealed a conserved principal oxidation pattern across the C2–C4 homologues, including +16 Da ions and two lower-mass ion series showing systematic 14 Da shifts with each additional methylene group, compatible with monooxygenation, oxidative ether cleavage, and *N*-dealkylation. In the **6c1**/**6c2** pair, 5-fluoro substitution removed the second-ranked isatin 5-position hydroxylation predicted for **6c1**, leaving the three highest-ranked predictions for **6c2** on the coumarin ring, and was accompanied by a selective 18 Da shift in an isatin-containing electrochemical oxidation product. **Conclusions:** The combined in silico and EC–MS approach identified structurally dependent predicted CYP-related properties and electrochemical oxidative susceptibility within this series. The findings support further evaluation of **6c1** and related analogues using biological metabolism models to establish their metabolic stability and CYP-mediated transformation pathways.

## 1. Introduction

Metabolic stability is an important consideration during lead optimization because the extent of biotransformation can influence drug exposure, clearance and duration of action [1,2]. Structural modifications introduced to improve biological activity may also alter metabolic stability or create new sites susceptible to enzymatic transformation [1]. Moreover, metabolism does not necessarily result only in drug inactivation or elimination. Some metabolites retain pharmacological activity [3], whereas others may undergo bioactivation to reactive species [4]. Identification of such metabolic liabilities at an early stage can therefore support the selection and further optimization of lead compounds.

Cytochrome P450 (CYP) enzymes are the major catalysts of oxidative drug metabolism [5]. Among them, CYP3A4, CYP2D6, CYP2C9, CYP2C19 and CYP1A2 contribute substantially to the metabolism of commonly used therapeutic agents [6,7]. Their reactions include hydroxylation, *N*- and *O*-dealkylation, heteroatom oxidation and dehydrogenation [5,7]. The site and extent of CYP-mediated oxidation depend on the physicochemical and structural properties of the substrate as well as its recognition and orientation within the enzyme active site [5,7]. Consequently, relatively small structural modifications may alter metabolic behaviour within a series of closely related compounds [5].

In silico approaches provide a rapid means of assessing CYP-related properties before experimental investigation. Current computational models can predict substrate or inhibitor interactions with individual CYP isoforms and assist in identifying potential metabolic liabilities [8,9]. Recent machine-learning approaches have also enabled the simultaneous evaluation of compounds against several major drug-metabolizing CYP isoforms [9,10,11]. However, prediction performance depends on the quality and composition of underlying experimental data and on how well the investigated structures are represented within the model applicability domain [8,11]. Computational predictions are therefore most useful as a guide for subsequent experimental investigation.

Electrochemical (EC) oxidation provides a complementary experimental approach for examining oxidative susceptibility. Controlled oxidation at a working electrode can generate transformation products that may be monitored by directly coupled mass spectrometry (MS) [12,13]. Product formation is influenced by factors including applied potential, electrode material and reaction medium, making optimization of experimental conditions important [12,13,14]. Although electrochemical systems can reproduce several transformations associated with phase I metabolism, the underlying electrochemical mechanisms do not necessarily correspond to those of enzyme-catalyzed oxidation [12,15]. Electrochemically generated products should therefore not automatically be considered biological metabolites, but they can reveal chemically accessible oxidative pathways and structural regions susceptible to oxidation.

EC–MS has also been applied to investigational bioactive compounds. For example, electrochemical oxidation combined with in silico prediction of CYP-mediated transformations has been reported for the antitumor-active imidazoacridinone C-1311 and the related triazoloacridinone C-1305 [16,17]. These studies illustrate the value of combining computational predictions with electrochemical experiments when examining the oxidative behaviour of individual compounds. Extending this complementary strategy to a structurally related analogue series additionally allows defined molecular modifications to be examined in relation to changes in oxidative susceptibility.

The coumarin–1,2,3-triazole–isatin hybrids investigated in the present study provide a suitable series for this purpose because their three constituent moieties have different structural and metabolic characteristics. Coumarin is a well-established CYP substrate, with CYP2A6-mediated 7-hydroxylation representing the principal oxidative pathway of the parent compound in humans [18]. In contrast, the 1,2,3-triazole ring is characterized by relatively high chemical and metabolic stability and is widely used as a bioisosteric motif in medicinal chemistry [19,20]. For the parent isatin scaffold, reduction of the C3 carbonyl group to 3-hydroxy-2-oxoindole has been identified as a human biotransformation pathway mediated predominantly by carbonyl reductase [21].

**Figure 1:**
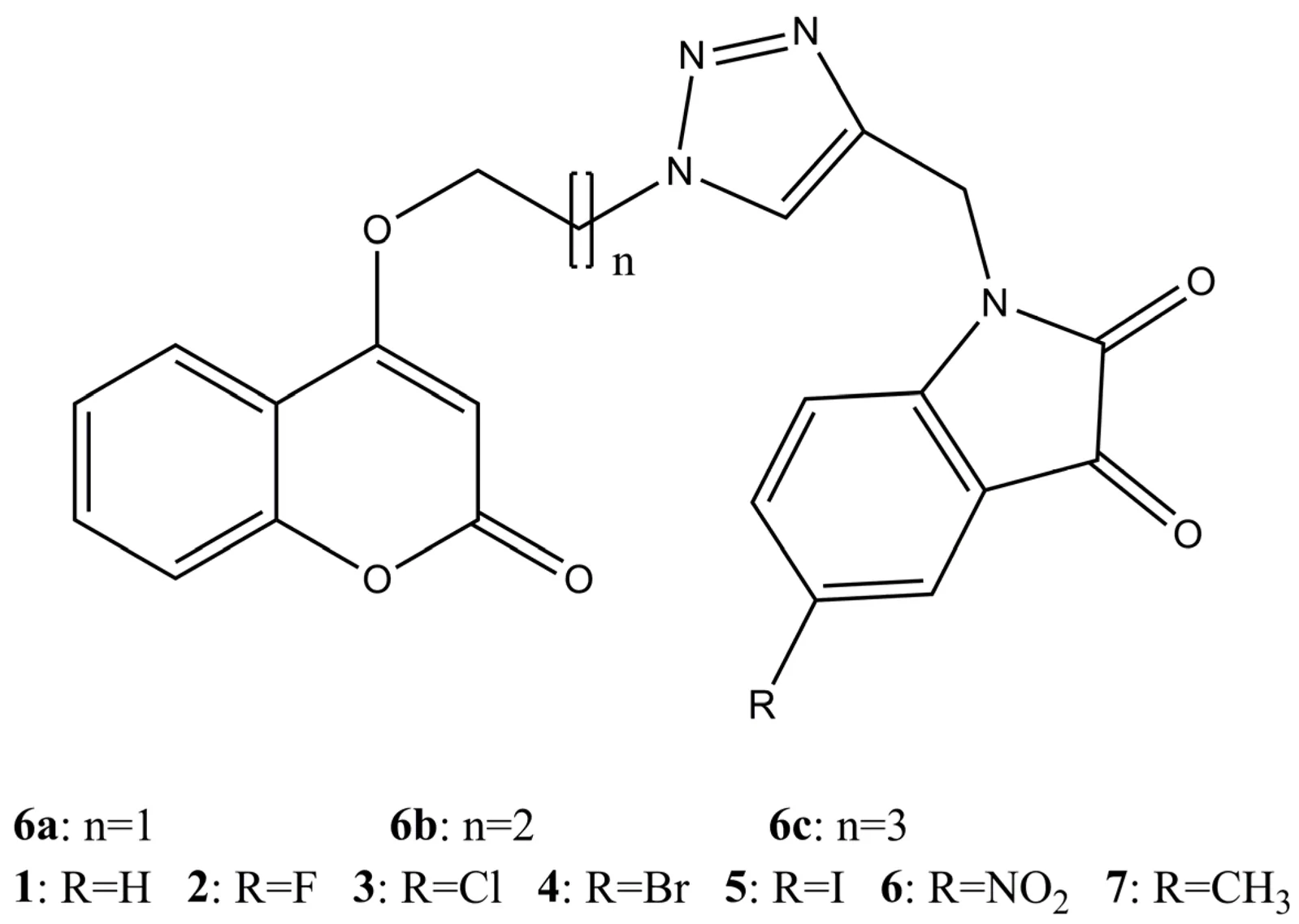
General structure of the coumarin–1,2,3-triazole–isatin hybrids investigated in the present study. Series **6a**, **6b** and **6c** contain two-, three- and four-carbon *O*-alkyl linkers, respectively. The substituent at the 5-position of the isatin ring is denoted by R (**1**, H; **2**, F; **3**, Cl; **4**, Br; **5**, I; **6**, NO_2_; **7**, CH_3_). Compounds **6a1**, **6b1**, **6c1** and **6c2** were selected for electrochemical oxidation.

We previously reported a series of 21 coumarin–1,2,3-triazole–isatin hybrids differing in the length of the *O*-alkyl linker connecting the coumarin and triazole moieties and in substitution at the 5-position of the isatin ring (Figure 1) [22]. The series comprises C2, C3, and C4 linker variants combined with H, F, Cl, Br, I, NO_2_, or CH_3_ substitution at the isatin 5-position. Several derivatives showed selective inhibition of butyrylcholinesterase (BChE), with compounds **6c1** and **6c2** among the most active members of the series [22]. Fluoro-substituted derivatives were generally more active than the corresponding analogues bearing the heavier halogens, whereas the 5-NO_2_ and 5-CH_3_-substituted analogues showed considerably lower activity [22]. The influence of fluorine and chlorine substitution on cholinesterase inhibition and selectivity has also been demonstrated in other compound series [23]. Despite the biological characterization of these hybrids, their oxidative behaviour has not previously been investigated.

In the present study, the complete 21-member series was evaluated in silico to obtain a series-wide assessment of CYP substrate and inhibitor profiles and predicted CYP-mediated transformations. Four structurally matched derivatives were then selected for online EC–MS analysis. Compounds **6a1**, **6b1**, and **6c1** form a homologous series in which only the *O*-alkyl linker length changes from two to four carbon atoms, allowing the influence of linker length to be examined while the remaining structural features are maintained. Compound **6c2** was included as the 5-fluoro analogue of **6c1**, providing a direct comparison of H and F substitution within the same C4 linker series. The study therefore combined series-wide computational assessment with targeted experimental comparison of two defined structural variables: linker length and 5-fluoro substitution.

The aim of the present work was to integrate computational CYP-related predictions with online EC–MS analysis to characterize the oxidative susceptibility of the coumarin–1,2,3-triazole–isatin hybrids and to determine whether the selected structural modifications were associated with differences in their oxidative profiles.

## 2. Results and Discussion

### 2.1. In Silico Assessment of CYP-Mediated Metabolism

The 21 coumarin–1,2,3-triazole–isatin derivatives were evaluated for CYP substrate and inhibitor profiles and for potential sites and pathways of oxidative metabolism. The predictions were used to compare isoform-related patterns across the series and to support interpretation of the subsequent EC–MS results. These CYP-related results are computational predictions and were not experimentally validated using recombinant CYP enzymes or microsomal systems. The EC–MS experiments were used separately to examine electrochemical oxidative susceptibility and should not be considered as direct evidence of CYP-mediated metabolism.

#### 2.1.1. Predicted CYP Substrate and Inhibitor Profile

CYPstrate (NERDD platform, University of Vienna, Austria) [24] showed an isoform-dependent pattern across the series (Table 1). All derivatives except the three 5-NO_2_ analogues, **6a6**, **6b6**, and **6c6**, were classified as CYP3A4 substrates, whereas these three compounds were non-predictable. For CYP2A6, the same three 5-NO_2_ derivatives were non-predictable, while all other compounds were classified as non-substrates. All derivatives were predicted to be non-substrates of CYP2B6 and CYP2E1. Predictions for the remaining isoforms were less complete: CYP1A2 non-substrate classifications were obtained for **6c1**, **6c2**, **6c3**, **6c4**, **6c5**, and **6c7**; CYP2C9 and CYP2D6 non-substrate classifications were obtained only for **6c6**, while no CYP2C19 classification was obtained for any derivative. Complete predictions and nearest-neighbour similarities are provided in Supplementary Table S1.

The CYP2A6 profile is of particular interest because CYP2A6-mediated 7-hydroxylation is the characteristic oxidative pathway of parent coumarin [18]. Incorporation of the coumarin moiety into the larger hybrid structure was associated with a different predicted CYP substrate profile, with CYP3A4 showing the most consistent substrate classification among compounds for which consensus predictions were obtained.

The non-predictable (NP) outputs require separate consideration. In the CYPstrate Best-performance mode, multiple classifiers are combined using a hard-voting consensus strategy, and no classification is returned when the required agreement among the component models is not achieved [24]. This approach improves classification performance at the expense of prediction coverage. Independent benchmarking of CYP prediction platforms has similarly shown that CYPstrate may leave a proportion of compounds unclassified because of limitations in model coverage [25]. Accordingly, NP results should not be interpreted as either substrate or non-substrate classifications.

This limitation is particularly relevant for CYP2C19, for which no substrate classification was obtained for any of the 21 derivatives. CYP2C19 is a clinically important and highly polymorphic drug-metabolizing enzyme [26]. Consequently, CYP2C19 substrate status remains unresolved for the present series, and the lack of a prediction does not provide evidence for or against CYP2C19 involvement.

The three 5-NO_2_ derivatives, **6a6**, **6b6** and **6c6**, were also non-predictable for both CYP3A4 and CYP2A6. Their CYP3A4 nearest-neighbour similarities were 0.73–0.75, indicating that the lack of a prediction is unlikely to be explained simply by poor structural similarity to the training set. The absence of consensus may instead reflect differences among the individual classifiers included in the CYPstrate model or other limitations of model applicability. Although all three nitro-substituted derivatives showed the same non-predictable outcome, the available data do not allow this to be linked specifically to the NO_2_ group. These three derivatives therefore remain unresolved with respect to CYP3A4 and CYP2A6 substrate classification, while the CYP3A4 pattern described above is based on the compounds for which a consensus prediction was obtained.

CYPlebrity (NERDD platform) [27] predicted low inhibition probabilities for CYP2D6 (0.26–0.36), whereas CYP3A4 probabilities ranged from 0.44 to 0.52. A probability of 0.50 was used as an operational cutoff for descriptive classification of the model output. The same threshold has been used in an independent comparison of CYP inhibition prediction tools, although the authors emphasized that different cutoffs may be selected depending on the desired balance between sensitivity and specificity [28]. Thus, values close to 0.50 should not be regarded as robust binary classifications.

For CYP3A4, the predicted probabilities clustered around this threshold, indicating considerable classification uncertainty. Nevertheless, the results do not support a consistent CYP3A4 inhibition across the series. This is relevant because CYP3A4 contributes substantially to the metabolism of many drugs and is frequently involved in clinically important drug–drug interactions [5,29,30].

For CYP1A2, CYP2C9 and CYP2C19, predicted inhibition probabilities ranged from 0.57–0.64, 0.54–0.59 and 0.58–0.64, respectively. Although these values were above the operational cutoff, they were only modestly above the decision boundary. False-positive classifications therefore cannot be excluded, and these predictions were interpreted cautiously as computational indications that require experimental confirmation.

Overall, the substrate predictions showed the clearest pattern for CYP3A4, although prediction coverage was incomplete for several other isoforms, particularly CYP2C8, CYP2C19, CYP2C9 and CYP2D6. In contrast, the inhibition predictions did not indicate a clear tendency toward CYP3A4 or CYP2D6 inhibition, while CYP1A2, CYP2C9 and CYP2C19 probabilities were only modestly above the operational cutoff and therefore remain uncertain. Given the clinical importance and genetic variability of CYP2D6 [31,32], its potential contribution to the metabolism of these compounds remains unresolved. Taken together, the results provide an initial CYP-related profile of the series but require experimental confirmation of both substrate status and inhibitory activity.

#### 2.1.2. Predicted Sites of Metabolism and CYP-Mediated Transformations

GLORY (NERDD platform) [33] generated ranked CYP-mediated transformations for each derivative, with aromatic hydroxylation emerging as the predominant top-ranked transformation across the series. Eighteen of the 21 compounds had aromatic hydroxylation as the first-ranked transformation, whereas the three 5-methyl derivatives (**6a7**, **6b7** and **6c7**) were predicted preferentially to undergo hydroxylation of the methyl substituent. Complete predictions are provided in Supplementary Table S3. Because GLORY predicts CYP-mediated transformation sites without assigning them to individual CYP isoforms, its results were interpreted separately from the isoform-specific CYPstrate classifications [33]. The GLORY rankings were used to compare predicted oxidative soft spots across the series and were not treated as definitive predictions of the metabolite profiles. Lower-ranked transformations were also considered because the GLORY ranking reflects their predicted likelihood in CYP-mediated metabolism and does not predict their relative formation or detectability under electrochemical conditions. Metabolic soft-spot identification can nevertheless provide useful guidance for structural optimization and subsequent experimental evaluation [34]. Comparison with additional independent platforms predicting the same endpoints could further assess the robustness of the present results, particularly for borderline or non-predictable cases.

**Figure 2:**
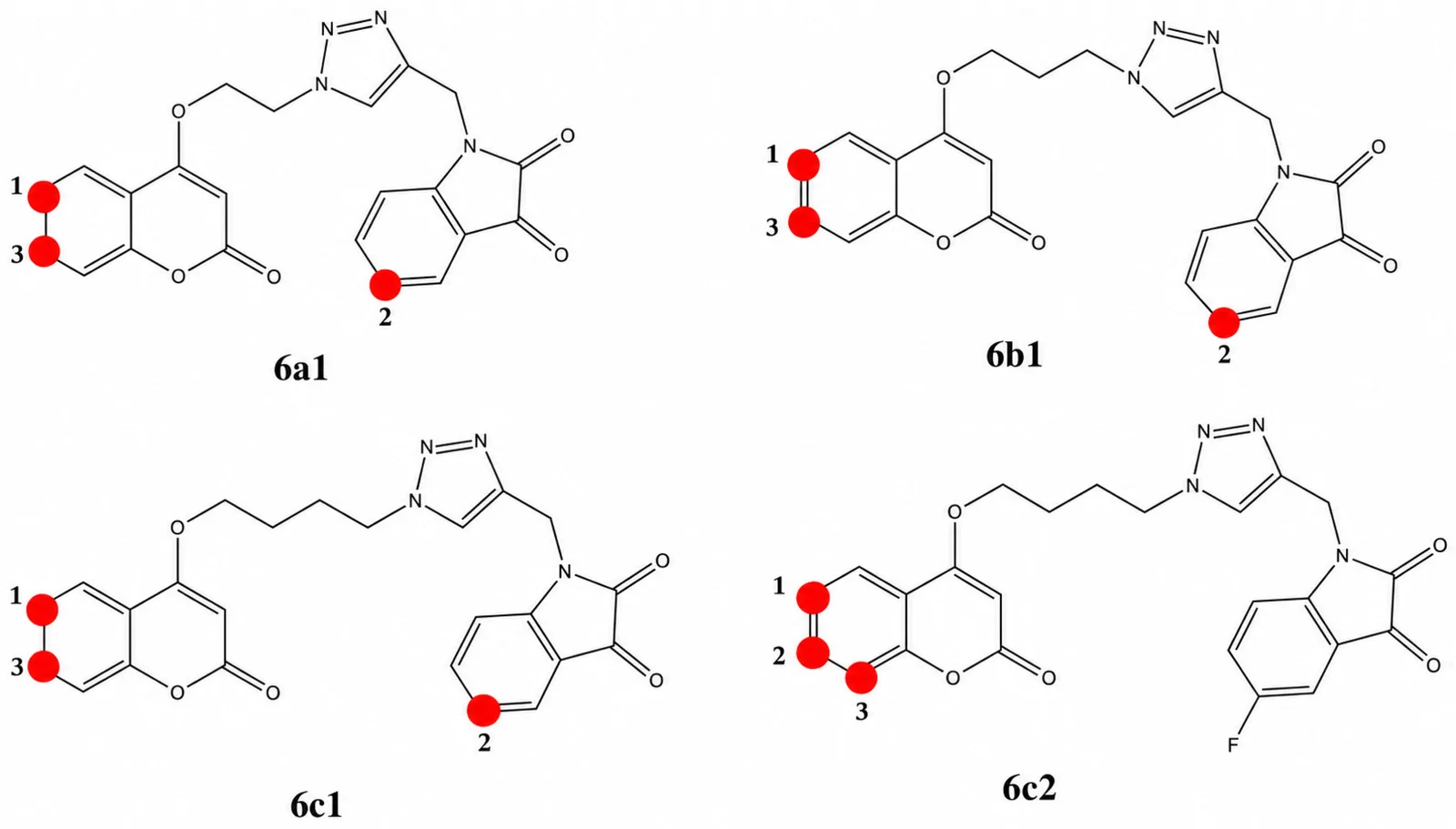
Three highest-ranked CYP-mediated oxidation sites (red circles) predicted by GLORY for compounds **6a1**, **6b1**, **6c1** and **6c2**. Numbers 1–3 indicate the rank of the predicted transformation, with 1 representing the highest-ranked site. For **6a1**, **6b1** and **6c1**, a coumarin-ring site is ranked first, the 5-position of the isatin ring second, and a second coumarin-ring site third. In **6c2**, the three highest-ranked sites are located on the coumarin ring. Numbers indicate GLORY rank.

For the three unsubstituted derivatives selected for electrochemical investigation, **6a1**, **6b1** and **6c1**, the same three aromatic hydroxylation sites occupied the highest positions irrespective of linker length (Figure 2). A coumarin-ring hydroxylation site was ranked first, hydroxylation at the 5-position of the isatin aromatic ring was ranked second, and a second coumarin-ring site was ranked third. Extension of the *O*-alkyl linker from two to four carbon atoms did not alter these principal predicted soft spots, although additional lower-ranked aliphatic hydroxylation and dehydrogenation pathways appeared with increasing linker length (Table S3). The predicted coumarin-ring hydroxylation does not conflict with the CYP2A6 non-substrate classifications obtained with CYPstrate, because GLORY does not assign this transformation to CYP2A6 specifically. Thus, incorporation of the coumarin moiety into the larger hybrid structure may alter isoform-level substrate recognition while retaining the coumarin ring as a potential site of CYP-mediated oxidative transformation.

For **6c2**, substitution at the isatin 5-position with fluorine removed the second-ranked 5-position C–H oxidation site predicted for **6c1**. Consequently, the three highest-ranked transformations for **6c2** were all coumarin-ring hydroxylations, while other positions of the isatin ring remained susceptible to lower-ranked aromatic hydroxylation. This redistribution is consistent with the established use of fluorine in medicinal chemistry to modify metabolic pathways and suppress oxidation at metabolically labile positions [35].

The GLORY predictions were used to identify possible CYP-mediated transformation sites and to support interpretation of the EC–MS results. However, the two approaches provide different types of structural information. GLORY predicts likely sites of metabolism, whereas the EC–MS experiments reveal oxidation-related ions and mass changes without directly establishing the position of the structural modification. The experimental findings were therefore interpreted separately from the predicted oxidation sites, and no specific site was considered experimentally confirmed. Further structural characterization by accurate-mass and tandem MS would strengthen the proposed product assignments, while unambiguous localization of the oxidation site may require isolation of the corresponding products followed by additional structural characterization, such as nuclear magnetic resonance spectroscopy (NMR) analysis.

### 2.2. Optimization of Electrochemical Oxidation Conditions

Electrochemical conditions were optimized using compound **6c1** as a representative derivative by varying the working-electrode material, reaction medium, electrolyte concentration, analyte concentration and applied potential. Cell-off spectra of **6c1** were used as reference measurements, while corresponding blank solutions were analyzed to identify background signals. Because the recorded MS responses represent ion signal intensities and not quantitative product concentrations, condition selection was based on comparative changes in the overall MS profile and the formation of oxidation-related signals.

**Figure 3:**
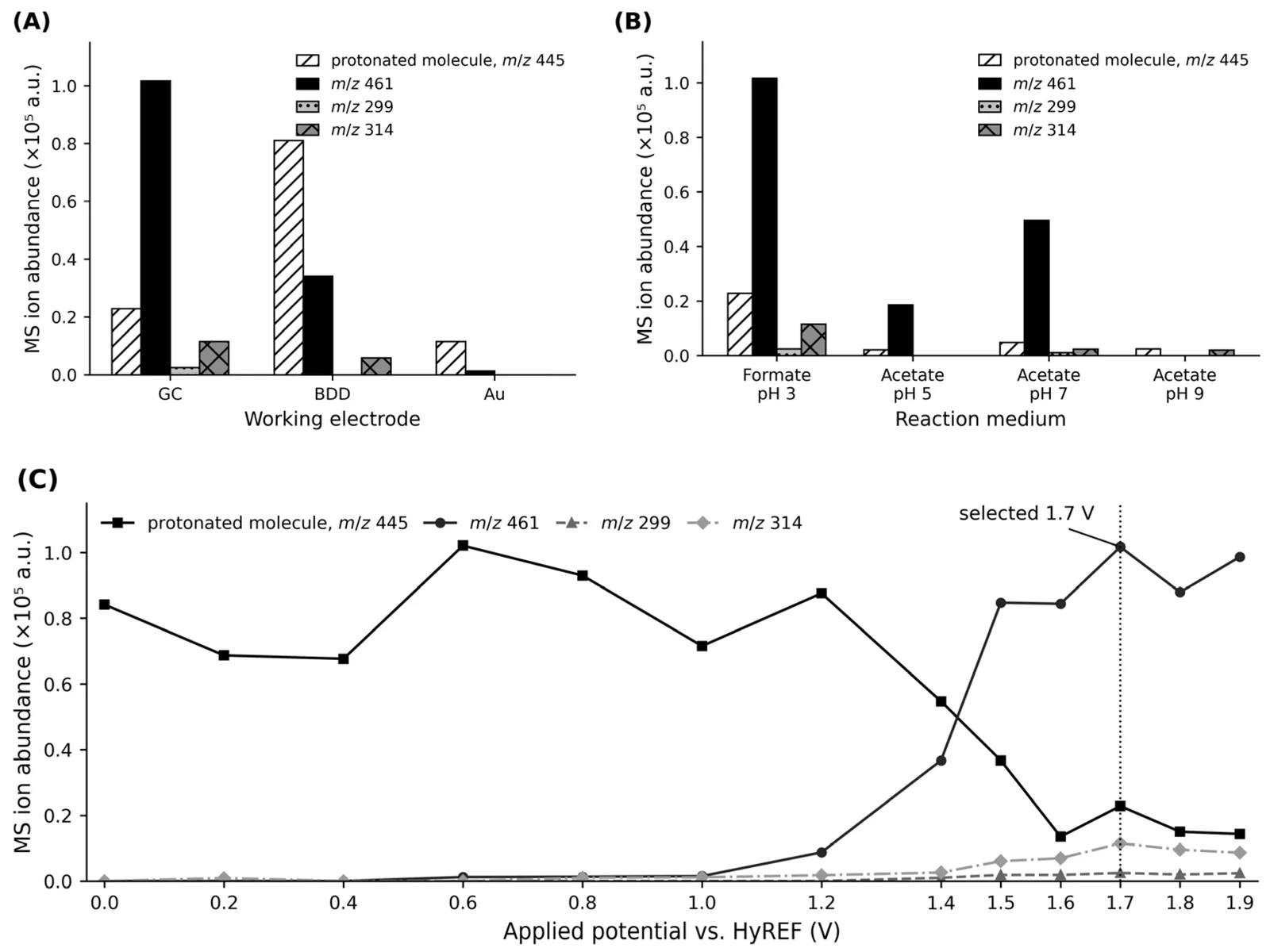
Optimization of electrochemical oxidation conditions for **6c1**. (**A**) Effect of working-electrode material and (**B**) reaction medium on the MS responses of **6c1** [M + H]^+^ at *m*/*z* 445 and the oxidation-related ions at *m*/*z* 461, 299 and 314. (**C**) Effect of applied potential on the same ions using the GC electrode in ammonium formate at pH 3.0. A potential of 1.7 V vs. HyREF was selected for subsequent experiments.

The influence of the working-electrode material was first evaluated using glassy carbon (GC), boron-doped diamond (Antec Scientific, Zoeterwoude, The Netherlands) and gold (Au) electrodes with 20 mmol L^−1^ ammonium formate at pH 3.0 and 10 μmol L^−1^ **6c1**. At 1.7 V vs. HyREF, the GC electrode generated the most pronounced oxidation-related signal at *m*/*z* 461, together with several additional transformation-related ions. In comparison, BDD produced a lower *m*/*z* 461 response and retained a relatively strong signal for the protonated molecule at *m*/*z* 445, whereas the corresponding oxidation-related response at the Au electrode was weak. GC therefore provided the most suitable oxidation profile for subsequent experiments and was selected for further optimization (Figure 3A and Figure S1A). The dependence of electrochemical product formation on working-electrode material is consistent with previous EC–MS studies showing that electrode selection can markedly influence the extent and pattern of oxidation products generated under otherwise comparable conditions [13,36].

The reaction medium was subsequently evaluated using the GC electrode and 10 μmol L^−1^ **6c1**. Ammonium formate at pH 3.0 was compared with ammonium acetate adjusted to pH 5.0, 7.0 and 9.0. Because both electrolyte composition and pH differed among these conditions, the observed effects were interpreted as differences between the tested reaction media and not as an isolated pH effect. Similar EC–MS optimization approaches have been reported previously, including the use of different volatile electrolyte systems across a broad pH range [37] and the comparison of ammonium formate at pH 3 with ammonium acetate at pH 5.0, 7.0 and 9.0 during electrochemical oxidation studies [13]. At 1.7 V, ammonium formate at pH 3.0 produced the most pronounced *m*/*z* 461 response, approximately twice that obtained with ammonium acetate at pH 7.0 and more than fivefold that observed at pH 5.0, whereas no corresponding signal was detected at pH 9.0. An additional prominent ion at *m*/*z* 483, consistent with a putative [M+K]^+^ adduct of **6c1**, was particularly evident in the acetate-containing media and was also present under cell-off conditions. Although potassium was not intentionally added to the experimental solutions, the pronounced medium-dependent response of this ion indicates that changes in the *m*/*z* 445 response alone cannot be interpreted directly as changes in the amount of unoxidized parent compound. Ammonium formate at pH 3.0 was therefore selected because it provided the strongest response of the principal oxidation-related ion at *m*/*z* 461 together with a suitable overall oxidation profile (Figure 3B and Figure S1B).

The influence of applied potential was examined in greater detail using the GC electrode and ammonium formate at pH 3.0. Only a weak *m*/*z* 461 response was observed at lower potentials, followed by a marked increase above approximately 1.2–1.4 V. Between 1.2 and 1.7 V, the response increased more than tenfold and reached its highest observed value at 1.7 V. The working potential was selected based on the overall MS profile and not only the *m*/*z* 461 response. Potential-dependent changes in the parent ion at *m*/*z* 445 and other oxidation-related ions were also considered. At 1.7 V, the investigated oxidation-related ions were clearly detected, whereas increasing the potential above 1.7 V did not provide a consistent improvement in the overall oxidation profile. Accordingly, 1.7 V vs. HyREF was selected for subsequent electrochemical oxidation experiments.

At pH 3.0 and 1.7 V, increasing the ammonium formate concentration from 10 to 20 mmol L^−1^ increased the *m*/*z* 461 response by approximately 20% and increased the responses of several additional oxidation-related ions. Increasing the **6c1** concentration from 5 to 10 μmol L^−1^ produced an approximately twofold increase in the *m*/*z* 461 signal and similarly increased the responses of several oxidation-related ions (Supplementary Figure S1C,D). On this basis, 20 mmol L^−1^ ammonium formate and 10 μmol L^−1^ **6c1** were used in subsequent experiments.

Based on the combined optimization experiments, a GC working electrode, 20 mmol L^−1^ ammonium formate at pH 3.0, an analyte concentration of 10 μmol L^−1^ and an applied potential of 1.7 V vs. HyREF were selected for subsequent electrochemical oxidation of compounds **6a1**, **6b1**, **6c1** and **6c2**. All four compounds produced detectable oxidation-related ions under these conditions, supporting their suitability for comparative EC–MS analysis of the investigated derivatives. The use of common electrochemical conditions for comparative evaluation of structurally related compounds has also been reported in previous EC–MS studies [38,39].

### 2.3. Electrochemical Oxidation and Predicted Transformations

**Figure 4:**
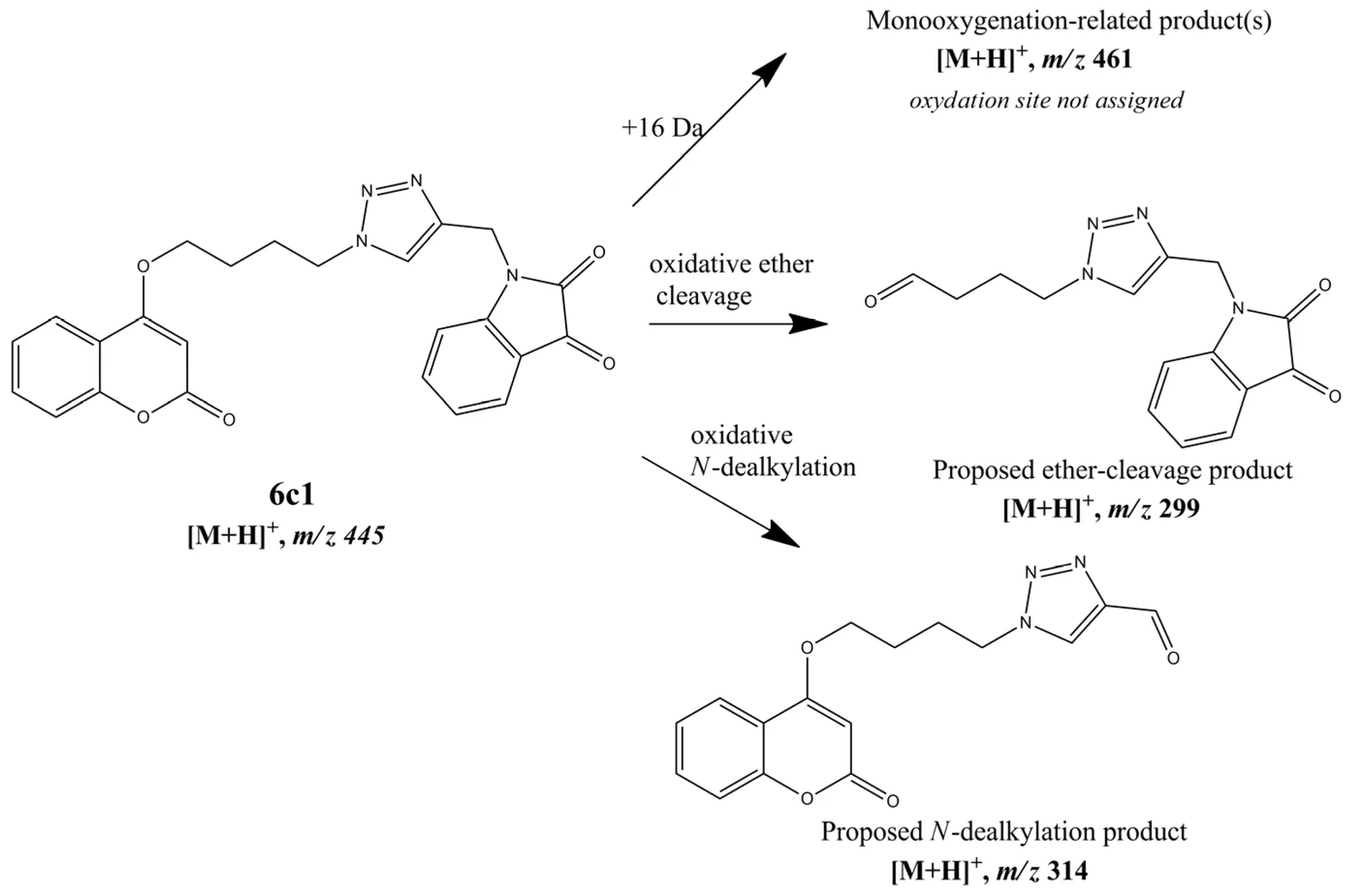
Proposed electrochemical transformations of **6c1** associated with the observed oxidation-related ions. The ion at *m*/*z* 461 corresponds to a +16 Da mass shift compatible with monooxygenation, while the ions at *m*/*z* 299 and 314 are consistent with oxidative ether cleavage and oxidative *N*-dealkylation, respectively.

Under the selected conditions, electrochemical oxidation of **6c1** resulted in a decrease in the response of protonated molecule at *m*/*z* 445 and the appearance of several oxidation-related ions (Figure 4). The most intense oxidation-related signal was observed at *m*/*z* 461, corresponding to a nominal mass increase of 16 Da. This mass shift is compatible with monooxygenation, which has also been reported in electrochemical models of oxidative drug metabolism [40,41]. Several oxidative transformations predicted for **6c1** could give the same nominal mass increase, including aromatic and aliphatic hydroxylation and *N*-oxidation. The EC–MS data therefore support the occurrence of an oxidation-related transformation but do not establish the specific oxidation type and site. The exact structure and position of the modification cannot be assigned from the present full-scan MS data.

Two lower-mass ions were also associated with electrochemical oxidation. The signal at *m*/*z* 299 was absent under cell-off conditions and appeared after application of the oxidation potential. Its nominal mass is consistent with a triazole–isatin-containing product formed by oxidative cleavage of the ether linkage. The complementary hydroxycoumarin product would be expected at approximately *m*/*z* 163; however, a signal in this region was also present in the blank and did not increase upon oxidation and was therefore not assigned as an electrochemically generated product.

A second oxidation-related ion at *m*/*z* 314 was likewise absent under cell-off conditions and increased markedly following electrochemical oxidation. Its nominal mass is compatible with the aldehyde-containing product proposed to arise from oxidative *N*-dealkylation of the isatin *N*-methylene linkage. The complementary isatin-derived product expected at approximately *m*/*z* 148 was not detected. The absence of these complementary products limits definitive assignment of the proposed pathways and may reflect differences in ionization efficiency under positive-ion ESI conditions, low product abundance, or further transformation of the initially formed species [12,42,43]. Electrochemical *N*-dealkylation has also been described for other drug-like compounds investigated by EC–MS [16,44].

The experimental findings therefore show both agreement with and extension of the highest-ranked GLORY predictions. The +16 Da product is compatible with the predominantly predicted monooxygenation pathways, whereas ether cleavage and *N*-dealkylation represent additional transformations that were also predicted by GLORY, although at lower ranks. For **6c1**, oxidative ether cleavage was ranked fifth and *N*-dealkylation twelfth (Table 2). Their detection by EC–MS does not imply that these pathways are as important as aromatic hydroxylation in CYP-mediated metabolism, since electrochemical product formation is governed by different reaction conditions and the observed MS response also depends on the ionization properties of the products under the applied conditions.

Application of the selected conditions to **6a1** and **6b1** produced oxidation profiles closely related to that of 6c1. The protonated molecules of **6a1**, **6b1** and **6c1** were observed at *m*/*z* 417, 431 and 445, respectively, while corresponding +16 Da ions occurred at *m*/*z* 433, 447 and 461 (Table 2). Thus, extension of the *O*-alkyl linker from two to four carbon atoms did not prevent formation of the principal +16 Da oxidation product. Although this pattern is compatible with monooxygenation in all three homologues, the common nominal mass shift does not establish that oxidation occurred at the same position in each compound. The +16 Da ions therefore indicate a shared type of oxidation-related transformation across the series, while the specific oxidation site remains unresolved from the present full-scan MS data.

The lower-mass oxidation products showed a clear homologous relationship. Signals at *m*/*z* 271, 285 and 299 were observed for 6a1, 6b1 and 6c1, respectively, corresponding to an increase of 14 Da with each additional methylene group in the linker. These ions are consistent with the corresponding triazole–isatin-containing products formed by oxidative ether cleavage. A second series was observed at *m*/*z* 286, 300 and 314. The same 14 Da progression is consistent with structurally related products arising from oxidative *N*-dealkylation and retaining the linker-derived portion of the molecule. Linker extension therefore changed the nominal masses of these products in the expected manner without markedly altering the overall oxidation profile.

The effect of substitution at the isatin 5-position was examined by comparison of **6c1** with the 5-fluoro analogue **6c2**. The protonated molecule of **6c2** was observed at *m*/*z* 463, and electrochemical oxidation produced a corresponding +16 Da ion at *m*/*z* 479. Introduction of fluorine therefore did not prevent formation of a monooxygenation-related product, although the present data do not permit localization of the oxidation site.

A more structurally informative difference was observed for the proposed ether-cleavage product. The corresponding ion shifted from *m*/*z* 299 for **6c1** to *m*/*z* 317 for **6c2**. The 18 Da increase corresponds to substitution of hydrogen by fluorine and is consistent with retention of the 5-fluoroisatin moiety in this product. In contrast, the aldehyde-containing ion associated with the proposed oxidative *N*-dealkylation pathway remained at *m*/*z* 314 for both **6c1** and **6c2**. This is consistent with cleavage of the isatin *N*-methylene bond and formation of a product that no longer contains the isatin moiety, explaining why fluorine substitution does not alter its nominal mass. These mass-shift patterns support the proposed product relationships, but they do not confirm the structures. The assignments therefore remain tentative and would require high-resolution mass spectrometry (HRMS) and tandem MS analysis for further confirmation.

The EC–MS experiments demonstrate that these compounds can undergo the observed oxidative transformations under the applied electrochemical conditions. However, this does not establish that the same products are formed during CYP-mediated metabolism in biological systems. Experimental assessment of metabolic stability is an important component of the broader pharmacokinetic characterization of bioactive compounds during their development [45]. The detected ions therefore provide experimental evidence of oxidative susceptibility and possible transformation pathways, while their relevance to human metabolism requires confirmation using human liver microsomes or recombinant CYP enzymes.

Overall, the +16 Da ions observed for all four investigated derivatives, together with the systematic 14 Da shifts across the homologous series and the selective 18 Da shift between **6c1** and **6c2**, support the proposed relationships among the electrochemically generated products. The experimentally observed ions are compatible with monooxygenation, oxidative ether cleavage and oxidative *N*-dealkylation predicted in silico. However, these products cannot be regarded as confirmed CYP metabolites, and the proposed structures remain tentative because the assignments are based on nominal-mass full-scan MS. Accurate-mass and tandem MS analyses would be required for structural confirmation.

## 3. Materials and Methods

### 3.1. Compounds and Reagents

The synthesis and structural characterization of the 21 coumarin–1,2,3-triazole–isatin derivatives investigated in the present study were reported previously [22]. All derivatives were evaluated in silico for CYP-related properties. Compounds **6a1**, **6b1**, **6c1** and **6c2** were selected for analysis by online EC–MS. Acetonitrile and water of LC–MS grade, formic acid, ammonium formate, ammonium acetate, acetic acid and aqueous ammonia suitable for LC–MS applications, together with dimethyl sulfoxide (DMSO) suitable for liquid chromatography, were obtained from Merck KGaA (Darmstadt, Germany).

### 3.2. In Silico Assessment of CYP-Mediated Metabolism

CYP-related properties were evaluated using the Next-generation E-Resource for Drug Discovery (NERDD) platform, developed by Univerity of Vienna, Austria. The structures of all 21 derivatives were submitted as SMILES strings. CYPstrate was used to predict substrate status for nine human CYP isoforms: CYP1A2, CYP2A6, CYP2B6, CYP2C8, CYP2C9, CYP2C19, CYP2D6, CYP2E1 and CYP3A4 [24]. Predictions were performed using the Best performance mode, which combines multiple classification models by majority voting; compounds were classified as substrates or non-substrates when a consensus prediction was available, while non-predictable cases were recorded accordingly. CYP inhibition was independently assessed using CYPlebrity for CYP1A2, CYP2C9, CYP2C19, CYP2D6 and CYP3A4 [27]. The five isoforms common to both approaches were considered when comparing predicted substrate and inhibitor profiles.

Potential CYP-mediated transformation pathways were further evaluated using GLORY [33]. The tool combines predicted sites of metabolism with CYP-mediated reaction rules to generate and rank plausible metabolite structures. Predictions were performed using the MaxCoverage mode, in which reaction rules are applied to all positions in the molecule and the resulting structures are subsequently ranked. The highest-ranked transformations were considered when comparing computational predictions with electrochemically generated oxidation products.

No experiments with recombinant CYP enzymes or microsomal systems were performed in the present study; the CYP-related assessments were based on in silico predictions.

### 3.3. Electrochemical Oxidation

Electrochemical oxidation was performed using a ROXY™ Exceed system (Antec Scientific, Alphen aan den Rijn, The Netherlands) equipped with a µ-PrepCell 2.0™ thin-layer flow-through electrochemical cell (Antec Scientific, Alphen aan den Rijn, The Netherlands) and a syringe infusion pump (Antec Scientific, Alphen aan den Rijn, The Netherlands). The system was controlled using Dialogue Elite software, version 2.21.8.1 (Antec Scientific). The µ-PrepCell 2.0™ was operated in a three-electrode configuration using interchangeable glassy carbon (GC), boron-doped diamond (BDD; Magic Diamond™) and gold (Au) working electrodes (Antec Scientific, Alphen aan den Rijn, The Netherlands), together with a HyREF™ (Pd/H_2_) reference electrode and a carbon-filled polytetrafluoroethylene (PTFE) auxiliary (Antec Scientific, Alphen aan den Rijn, The Netherlands). Two 50 μm spacers were used, corresponding to a cell volume of approximately 20 μL and a wetted working-electrode area of 1.9 cm^2^. The cell temperature was maintained at 37 °C throughout the experiments.

Working-electrode handling and maintenance followed the manufacturer’s recommendations for the µ-PrepCell 2.0™. Electrode surfaces were inspected before use and cleaned when necessary to minimize surface fouling. Mechanical polishing was avoided for the boron-doped diamond electrode, in accordance with the manufacturer’s instructions. Between measurements, the µ-PrepCell 2.0™ was flushed with fresh reaction medium before introduction of the subsequent sample.

Stock solutions of compounds **6a1**, **6b1**, **6c1** and **6c2** (0.5 mmol L^−1^) were prepared in DMSO. Initial working solutions (10 μmol L^−1^) contained 24.5% (*v*/*v*) acetonitrile and 2.0% (*v*/*v*) DMSO in the corresponding aqueous ammonium formate or ammonium acetate solution and were infused through the µ-PrepCell 2.0™ at a flow rate of 20 μL min^−1^. Blank solutions containing the same proportions of acetonitrile and DMSO and the corresponding electrolyte, but without the test compound, were analyzed to identify solvent- and matrix-related background signals. Signals present in the blank were not assigned as electrochemically generated products, while oxidation-related ions were identified from their appearance or increase following application of the electrochemical potential.

Optimization of the electrochemical conditions was performed using compound **6c1** as a representative derivative. The influence of working-electrode material and reaction medium was initially investigated using GC, BDD and Au electrodes in combination with ammonium formate at pH 3.0 and ammonium acetate at pH 5.0, 7.0 and 9.0. Following this initial screening, further optimization was performed using the GC electrode and ammonium formate at pH 3.0. Ammonium formate concentrations of 10 and 20 mmol L^−1^ and analyte concentrations of 5 and 10 μmol L^−1^ were investigated.

The influence of applied potential was also evaluated using **6c1** in Direct Current (DC) mode, with a constant potential applied during each individual measurement. For GC and Au electrodes, potentials from 0.0 to 2.0 V vs. HyREF were investigated, whereas the potential range for the BDD electrode was extended to 3.0 V vs. HyREF. For all electrodes, potentials from 0.0 to 1.4 V were applied at 0.2-V intervals, followed by 0.1-V intervals above 1.4 V up to the respective maximum potential. At each applied potential, the µ-PrepCell 2.0™ effluent was introduced directly into the electrospray ionization source of the mass spectrometer for online electrochemistry–mass spectrometry (EC–MS) analysis. Measurements with the µ-PrepCell 2.0™ switched off (cell-off condition) were acquired as controls to establish the MS profile of the unoxidized compound and to distinguish electrochemically generated products from ions already present in the solution or formed independently of the applied potential.

Subsequent electrochemical oxidation experiments with compounds **6a1**, **6b1**, **6c1** and **6c2** were performed using a GC working electrode, 20 mmol L^−1^ ammonium formate at pH 3.0, an analyte concentration of 10 μmol L^−1^ and an applied potential of 1.7 V vs. HyREF. Electrochemical oxidation experiments under the selected conditions were performed in triplicate for each compound.

### 3.4. Mass Spectrometric Analysis

Mass spectrometric detection was performed using an Ultivo triple quadrupole mass spectrometer (G6465BA, Agilent Technologies, Inc., Santa Clara, CA, USA) equipped with an electrospray ionization (ESI) source. Spectra were acquired in full-scan mode using positive-ion electrospray ionization over an *m*/*z* range of 100–550. Positive-ion ESI was selected based on the ionization behaviour observed during the previous characterization of these compounds, in which the protonated molecular ions were readily detected, whereas the response in negative-ion mode was weak [22]. Although the parent compounds showed weak ionization in negative-ion mode, complementary negative-ion analysis may still be useful for detecting oxidation products with different ionization properties and for extending the characterization of the electrochemically generated product profile. The ESI source parameters were as follows: capillary voltage, 4000 V; drying-gas temperature, 320 °C; drying-gas flow, 11 L min^−1^; and nebulizer pressure, 35 psi. The same MS conditions were maintained throughout the electrochemical experiments. Data acquisition and instrument control were performed using Agilent MassHunter Workstation LC/MS Data Acquisition for Ultivo LC/TQ, version 1.2 (Agilent Technologies), while mass-spectral data were evaluated using Agilent MassHunter Workstation Qualitative Analysis, version 10.0 (Agilent Technologies).

Electrochemically generated oxidation-related ions were identified from their appearance upon application of potential and from their mass differences relative to the corresponding parent compounds. For **6c1**, potential-dependent changes in the responses of the protonated molecule and oxidation-related ions were additionally used during optimization of the electrochemical conditions. Observed mass shifts were compared with the transformations predicted in silico. The present full-scan MS data provide evidence for the occurrence of the proposed oxidative transformations but do not permit complete structural characterization of the products. Further characterization by HRMS could establish accurate elemental compositions, while MS/MS could provide diagnostic fragmentation patterns supporting the proposed structures. Since several oxidation pathways may generate positional isomers with the same elemental composition, definitive localization of the structural change may require isolation of the electrochemically generated products followed by NMR analysis, especially in the absence of authentic metabolite standards.

## 4. Conclusions

In this study, in silico assessment of CYP-related properties and online EC–MS were used to investigate the oxidative behaviour of coumarin–1,2,3-triazole–isatin hybrids. The combined approach identified several possible electrochemically accessible oxidative pathways and showed how structural changes within the series influence their electrochemical oxidation profiles.

The present findings should be interpreted within the limits of the methods used. The structures of the electrochemically generated products remain tentative because they were assigned from nominal-mass full-scan MS data. In addition, CYP substrate and inhibition profiles were predicted computationally and were not confirmed experimentally, while EC–MS does not establish that the observed products are formed through CYP-mediated metabolism. The metabolic stability of the investigated compounds was also not determined experimentally.

Further studies should therefore include human liver microsomes and recombinant CYP enzymes to evaluate metabolic stability and establish the contribution of individual CYP isoforms. Characterization of the metabolites by LC–HRMS/MS would provide stronger structural evidence and allow comparison of biological and electrochemical oxidation pathways. Where positional isomers cannot be distinguished by mass spectrometry alone, isolation and NMR characterization may provide additional structural confirmation. These studies will be particularly important for **6c1** and related active analogues before further development.

## Figures and Tables

**Table 1:** Predicted CYP substrate and inhibitor profiles of the coumarin–1,2,3-triazole–isatin derivatives.

CYP Isoform	CYPstrate	CYPlebrity	
	Substrate Classification	Inhibition Probability	Compounds with Probability ≥ 0.50
CYP1A2	NS: **6c1**, **6c2**, **6c3**, **6c4**, **6c5**, **6c7**; others NP	0.57–0.64	All derivatives
CYP2A6	NS: all except **6a6**, **6b6** and **6c6**; these NP	NE	–
CYP2C9	NS: **6c6**; others NP	0.54–0.59	All derivatives
CYP2C19	NP: all derivatives	0.58–0.64	All derivatives
CYP2D6	NS: **6c6**; others NP	0.26–0.36	None
CYP3A4	S: all except **6a6**, **6b6** and **6c6**; these NP	0.44–0.52	**6a5**, **6a7**, **6c2**, **6c5**, **6c7**

*S*—substrate; NS—non-substrate; NP—no prediction; NE—not evaluated by CYPlebrity. Additional CYPstrate predictions: all derivatives were classified as non-substrates of CYP2B6 and CYP2E1, whereas no CYP2C8 classification was obtained for any derivative. CYPlebrity values represent predicted inhibition probabilities; a probability of 0.50 was used as an operational cutoff for descriptive classification; values close to this threshold were considered uncertain and were not interpreted as evidence of confirmed CYP inhibition.

**Table 2:** Selected EC–MS ions and their relationship to GLORY-predicted transformations.

Compound	Observed EC–MS Data			Compatibility with GLORY Predicted Transformations
	Parent Ion, *m*/*z* [M+H]^+^	Detected Ion, *m*/*z*	Nominal Mass Change	
**6a1**	417	433	+16 Da	Monooxygenation; compatible with GLORY-predicted hydroxylation ^a^
		271	−146 Da	Compatible with GLORY-predicted oxidative ether cleavage
		286	−131 Da	Compatible with GLORY-predicted oxidative *N*-dealkylation
**6b1**	431	447	+16 Da	Monooxygenation; compatible with GLORY-predicted hydroxylation ^a^
		285	−146 Da	Compatible with GLORY-predicted oxidative ether cleavage
		300	−131 Da	Compatible with GLORY-predicted oxidative *N*-dealkylation
**6c1**	445	461	+16 Da	Monooxygenation; compatible with GLORY-predicted hydroxylation ^a^
		299	−146 Da	Compatible with GLORY-predicted oxidative ether cleavage ^b^
		314	−131 Da	Compatible with GLORY-predicted oxidative *N*-dealkylation ^c^
**6c2**	463	479	+16 Da	Monooxygenation; compatible with GLORY-predicted hydroxylation ^d^
		317	−146 Da	Compatible with GLORY-predicted oxidative ether cleavage
		314	−149 Da	Compatible with GLORY-predicted oxidative *N*-dealkylation

^a^ For **6a1**, **6b1** and **6c1**, coumarin-ring hydroxylation was the highest-ranked GLORY transformation, followed by hydroxylation at the 5-position of the isatin ring and a second coumarin-ring hydroxylation. ^b^ Oxidative ether cleavage was ranked fifth by GLORY for **6c1**. ^c^ Oxidative *N*-dealkylation was ranked twelfth by GLORY for 6c1. ^d^ For **6c2**, the three highest-ranked aromatic hydroxylation predictions were located on the coumarin ring; fluorination removed the high-ranked isatin 5-position hydroxylation predicted for **6c1**. Assignments are tentative and are based on nominal mass differences, homologous mass-shift patterns and comparison with GLORY predictions; tandem MS experiments were not performed.

## Data Availability

The original contributions presented in this study are included in the article/Supplementary Material. Further inquiries can be directed to the corresponding author.

## Supplementary Materials

The following supporting information can be downloaded at: https://www.mdpi.com/article/10.3390/ph19101568/s1, Table S1: CYPstrate predictions and nearest-neighbour similarities for the coumarin–1,2,3-triazole–isatin derivatives; Table S2: CYPlebrity-predicted inhibition probabilities and nearest-neighbour similarities for the coumarin–1,2,3-triazole–isatin derivatives; Table S3: Ranked CYP-mediated transformations predicted by GLORY for the coumarin–1,2,3-triazole–isatin derivatives; Figure S1: Full-scan positive-ion ESI mass spectra supporting optimization of electrochemical oxidation conditions for **6c1**.

## Abbreviations

The following abbreviations are used in this manuscript:

Au	Gold
BDD	Boron-doped diamond
BChE	Butyrylcholinesterase
CYP	Cytochrome P450
DC	Direct current
DMSO	Dimethyl sulfoxide
EC-MS	Electrochemistry–mass spectrometry
ESI	Electrospray ionization
GC	Glassy carbon
HRMS	High-resolution mass spectrometry
LC-HRMS/MS	Liquid chromatography–high-resolution tandem mass spectrometry
LC-MS	Liquid chromatography–mass spectrometry
MS	Mass spectrometry
NERDD	Next-generation E-Resource for Drug Discovery
NMR	Nuclear magnetic resonance
PTFE	Polytetrafluoroethylene
SMILES	Simplified molecular-input line-entry system
*m*/*z*	Mass-to-charge ratio
