# Supplementary material for: Oxidative Susceptibility of Coumarin–1,2,3-Triazole–Isatin Hybrids: Integrating In Silico CYP-Mediated Metabolism Prediction with Electrochemistry–Mass Spectrometry
Source: Pharmaceuticals (Basel). 2026 Oct 1;19(10):1568. doi: 10.3390/ph19101568 (PMC13708526; doi:10.3390/ph19101568)
Supplement: Supplementary file 1 [file pharmaceuticals-19-01568-s001.zip › pharmaceuticals-4585617-supplementary.pdf]

PHARMACEUTICALS

Supplementary Materials associated with the paper

Oxidative Susceptibility of Coumarin–1,2,3-Triazole–Isatin Hybrids: Integrating In Silico CYP-Mediated Metabolism Prediction with Electrochemistry–Mass Spectrometry

Aleksandar Dimkovski [1*], Darija Krzhovska [1], Vladimir Dobričić [2], Milena R. Simić [3], Evgenija Mihajloska [1], Aleksandra Kapedanovska Nestorovska [1], Aleksandra Grozdanova [1], Ana Poceva Panovska [4] and Zoran Sterjev [1]

[1] Institute for Pharmaceutical Chemistry, Faculty of Pharmacy, Ss Cyril and Methodius University in Skopje, Majka Tereza 47, 1000 Skopje, North Macedonia; darija@ff.ukim.edu.mk (D.K.); emihajloska@ff.ukim.edu.mk (E.M.); alka@ff.ukim.edu.mk (A.K.N.); agrozdanova@ff.ukim.edu.mk (A.G.); zost@ff.ukim.edu.mk (Z.S.)

[2] Department of Pharmaceutical Chemistry, Faculty of Pharmacy, University of Belgrade, Vojvode Stepe 450, 11221 Belgrade, Serbia; vladimir.dobricic@pharmacy.bg.ac.rs

[3] Department of Organic Chemistry, Faculty of Pharmacy, University of Belgrade, Vojvode Stepe 450, 11221 Belgrade, Serbia; milena.simic@pharmacy.bg.ac.rs

[4] Institute for Applied Chemistry and Pharmaceutical Analysis, Faculty of Pharmacy, Ss Cyril and Methodius University in Skopje, Majka Tereza 47, 1000 Skopje, North Macedonia; anpo@ff.ukim.edu.mk

* Correspondence: aleksandar.d@ff.ukim.edu.mk

Contents

Table S1. CYPstrate predictions and nearest-neighbour similarities for the coumarin–1,2,3-triazole–isatin derivatives 3

Table S2. CYPlebrity-predicted inhibition probabilities and nearest-neighbour similarities for the coumarin–1,2,3-triazole–isatin derivatives.......... 4

Table S3. Ranked CYP-mediated transformations predicted by GLORY for the coumarin–1,2,3-triazole–isatin derivatives 5–28

Figure S1. Full-scan positive-ion ESI mass spectra supporting optimization of electrochemical oxidation conditions for 6c1 29–31

Table S1. CYPstrate predictions and nearest-neighbour similarities for the coumarin–1,2,3-triazole–isatin derivatives

Compound	CYP1A2	CYP2A6	CYP2B6	CYP2C8	CYP2C9	CYP2C19	CYP2D6	CYP2E1	CYP3A4
**6a1**	NP (0.75)	NS (0.75)	NS (0.72)	NP (0.75)	NP (0.75)	NP (0.75)	NP (0.75)	NS (0.75)	S (0.72)
**6a2**	NP (0.78)	NS (0.78)	NS (0.70)	NP (0.78)	NP (0.78)	NP (0.78)	NP (0.78)	NS (0.78)	S (0.71)
**6a3**	NP (0.80)	NS (0.80)	NS (0.72)	NP (0.80)	NP (0.80)	NP (0.80)	NP (0.80)	NS (0.80)	S (0.72)
**6a4**	NP (0.78)	NS (0.78)	NS (0.70)	NP (0.78)	NP (0.78)	NP (0.78)	NP (0.78)	NS (0.78)	S (0.70)
**6a5**	NP (0.78)	NS (0.78)	NS (0.70)	NP (0.78)	NP (0.78)	NP (0.78)	NP (0.78)	NS (0.78)	S (0.70)
**6a6**	NP (0.70)	NP (0.70)	NS (0.68)	NP (0.70)	NP (0.70)	NP (0.70)	NP (0.70)	NS (0.70)	NP (0.73)
**6a7**	NP (0.76)	NS (0.76)	NS (0.73)	NP (0.76)	NP (0.76)	NP (0.76)	NP (0.76)	NS (0.76)	S (0.74)
**6b1**	NP (0.75)	NS (0.75)	NS (0.72)	NP (0.75)	NP (0.75)	NP (0.75)	NP (0.75)	NS (0.75)	S (0.72)
**6b2**	NP (0.78)	NS (0.78)	NS (0.70)	NP (0.78)	NP (0.78)	NP (0.78)	NP (0.78)	NS (0.78)	S (0.71)
**6b3**	NP (0.80)	NS (0.80)	NS (0.72)	NP (0.80)	NP (0.80)	NP (0.80)	NP (0.80)	NS (0.80)	S (0.72)
**6b4**	NP (0.78)	NS (0.78)	NS (0.70)	NP (0.78)	NP (0.78)	NP (0.78)	NP (0.78)	NS (0.78)	S (0.70)
**6b5**	NP (0.78)	NS (0.78)	NS (0.70)	NP (0.78)	NP (0.78)	NP (0.78)	NP (0.78)	NS (0.78)	S (0.70)
**6b6**	NP (0.70)	NP (0.70)	NS (0.68)	NP (0.70)	NP (0.70)	NP (0.70)	NP (0.70)	NS (0.70)	NP (0.73)
**6b7**	NP (0.76)	NS (0.76)	NS (0.73)	NP (0.76)	NP (0.76)	NP (0.76)	NP (0.76)	NS (0.76)	S (0.74)
**6c1**	NS (0.76)	NS (0.76)	NS (0.74)	NP (0.76)	NP (0.76)	NP (0.76)	NP (0.76)	NS (0.76)	S (0.74)
**6c2**	NS (0.79)	NS (0.79)	NS (0.71)	NP (0.79)	NP (0.79)	NP (0.79)	NP (0.79)	NS (0.79)	S (0.72)
**6c3**	NS (0.81)	NS (0.81)	NS (0.73)	NP (0.81)	NP (0.81)	NP (0.81)	NP (0.81)	NS (0.81)	S (0.73)
**6c4**	NS (0.79)	NS (0.79)	NS (0.71)	NP (0.79)	NP (0.79)	NP (0.79)	NP (0.79)	NS (0.79)	S (0.71)
**6c5**	NS (0.79)	NS (0.79)	NS (0.71)	NP (0.79)	NP (0.79)	NP (0.79)	NP (0.79)	NS (0.79)	S (0.71)
**6c6**	NP (0.71)	NP (0.71)	NS (0.68)	NP (0.71)	NS (0.71)	NP (0.71)	NS (0.71)	NS (0.71)	NP (0.75)
**6c7**	NS (0.77)	NS (0.77)	NS (0.74)	NP (0.77)	NP (0.77)	NP (0.77)	NP (0.77)	NS (0.77)	S (0.75)

S – substrate; NS – non-substrate; NP – no prediction. Values in parentheses are nearest-neighbour Tanimoto similarities reported by CYPstrate.

Table S2. CYPlebrity-predicted inhibition probabilities and nearest-neighbour similarities for the coumarin–1,2,3-triazole–isatin derivatives

Compoun d	CYP1A2	CYP2C9	CYP2C19	CYP2D6	CYP3A4
**6a1**	0.64 (0.69)	0.56 (0.69)	0.64 (0.69)	0.32 (0.69)	0.47 (0.78)
**6a2**	0.57 (0.77)	0.57 (0.80)	0.61 (0.77)	0.29 (0.77)	0.49 (0.78)
**6a3**	0.59 (0.77)	0.59 (0.79)	0.62 (0.77)	0.32 (0.77)	0.48 (0.79)
**6a4**	0.58 (0.77)	0.58 (0.81)	0.60 (0.77)	0.31 (0.77)	0.47 (0.79)
**6a5**	0.58 (0.77)	0.57 (0.81)	0.59 (0.77)	0.32 (0.77)	0.50 (0.79)
**6a6**	0.57 (0.78)	0.56 (0.78)	0.63 (0.78)	0.26 (0.78)	0.44 (0.78)
**6a7**	0.59 (0.76)	0.58 (0.80)	0.64 (0.76)	0.28 (0.76)	0.50 (0.78)
**6b1**	0.64 (0.70)	0.55 (0.70)	0.61 (0.70)	0.33 (0.70)	0.46 (0.76)
**6b2**	0.58 (0.77)	0.56 (0.80)	0.58 (0.77)	0.33 (0.77)	0.48 (0.76)
**6b3**	0.59 (0.77)	0.58 (0.79)	0.62 (0.77)	0.35 (0.77)	0.47 (0.77)
**6b4**	0.60 (0.77)	0.56 (0.81)	0.59 (0.77)	0.33 (0.77)	0.46 (0.77)
**6b5**	0.58 (0.77)	0.57 (0.81)	0.58 (0.77)	0.34 (0.77)	0.49 (0.77)
**6b6**	0.58 (0.78)	0.54 (0.79)	0.60 (0.78)	0.30 (0.78)	0.44 (0.78)
**6b7**	0.60 (0.76)	0.57 (0.80)	0.61 (0.76)	0.30 (0.76)	0.48 (0.77)
**6c1**	0.64 (0.71)	0.55 (0.71)	0.62 (0.71)	0.33 (0.71)	0.49 (0.75)
**6c2**	0.57 (0.77)	0.56 (0.81)	0.60 (0.77)	0.33 (0.77)	0.51 (0.75)
**6c3**	0.58 (0.77)	0.58 (0.79)	0.62 (0.77)	0.36 (0.77)	0.49 (0.76)
**6c4**	0.59 (0.77)	0.58 (0.82)	0.61 (0.77)	0.33 (0.77)	0.48 (0.76)
**6c5**	0.57 (0.77)	0.57 (0.82)	0.59 (0.77)	0.35 (0.77)	0.51 (0.76)
**6c6**	0.57 (0.78)	0.56 (0.80)	0.62 (0.78)	0.29 (0.78)	0.46 (0.77)
**6c7**	0.59 (0.76)	0.57 (0.81)	0.64 (0.76)	0.31 (0.76)	0.52 (0.76)

Values are predicted inhibition probabilities, with nearest-neighbour (NN) Tanimoto similarities shown in parentheses. CYPlebrity probabilities represent the fraction of decision trees voting for the inhibitor class. A probability of 0.50 was used as the decision threshold for descriptive classification.

Table S3. Ranked CYP-mediated transformations predicted by GLORY for the coumarin–1,2,3-triazole–isatin derivatives

Compound	Rank	Priority score	Predicted transformation	Predicted metabolite SMILES
**Series 6a**				
**6a1 — Parent SMILES:** O=C1C(=O)N(Cc2cn(CCOC3=CC(=O)Oc4ccccc34)nn2)c2ccccc21				
**6a1**	1	2.730	aromatic hydroxylation	O=C1C(=O)N(Cc2cn(CCOc3cc(=O)oc4cc(O)ccc34)nn2)c2ccccc21
**6a1**	2	2.520	aromatic hydroxylation	O=C1C(=O)N(Cc2cn(CCOc3cc(=O)oc4ccccc34)nn2)c2ccc(O)cc21
**6a1**	3	1.610	aromatic hydroxylation	O=C1C(=O)N(Cc2cn(CCOc3cc(=O)oc4ccc(O)cc34)nn2)c2ccccc21
**6a1**	4	1.309	aromatic hydroxylation	O=C1C(=O)N(Cc2cn(CCOc3cc(=O)oc4c(O)cccc34)nn2)c2ccccc21
**6a1**	5	1.150	aromatic hydroxylation	O=C1C(=O)N(Cc2cn(CCOc3cc(=O)oc4ccccc34)nn2)c2c(O)cccc21
**6a1**	6	0.955	oxidative ether cleavage to one alcohol and one aldehyde/ketone	O=c1cc(O)c2ccccc2o1
**6a1**	6	0.955	aliphatic hydroxylation	O=C1C(=O)N(Cc2cn(CC(O)Oc3cc(=O)oc4ccccc34)nn2)c2ccccc21
**6a1**	6	0.955	oxidative ether cleavage to one alcohol and one aldehyde/ketone	O=CCn1cc(CN2C(=O)C(=O)c3ccccc32)nn1
**6a1**	9	0.860	aromatic hydroxylation	O=C1C(=O)N(Cc2cn(CCOc3cc(=O)oc4cccc(O)c34)nn2)c2ccccc21
**6a1**	10	0.790	aromatic hydroxylation	O=C1C(=O)N(Cc2cn(CCOc3cc(=O)oc4ccccc34)nn2)c2cc(O)ccc21
**6a1**	11	0.700	*N*-dealkylation	O=Cc1cn(CCOc2cc(=O)oc3ccccc23)nn1
**6a1**	11	0.700	aliphatic hydroxylation	O=C1C(=O)N(C(O)c2cn(CCOc3cc(=O)oc4ccccc34)nn2)c2ccccc21
**6a1**	11	0.700	*N*-oxidation	O=C1C(=O)[N+]([O-])(Cc2cn(CCOc3cc(=O)oc4ccccc34)nn2)c2ccccc21
**6a1**	11	0.700	*N*-dealkylation	O=C1Nc2ccccc2C1=O
**6a1**	15	0.650	aromatic hydroxylation	O=C1C(=O)N(Cc2cn(CCOc3cc(=O)oc4ccccc34)nn2)c2cccc(O)c21
**6a1**	16	0.546	oxidation of 4-substituted anisole to quinone	O=CC=C(OCCn1cc(CN2C(=O)C(=O)c3ccccc32)nn1)C1=CC(=O)C=CC1=O
**6a1**	17	0.460	*N*-oxidation	O=C1C(=O)N(Cc2cn(CCOc3cc(=O)oc4ccccc34)n[n+]2[O-])c2ccccc21
**6a1**	18	0.270	*N*-oxidation	O=C1C(=O)N(CC2=C[N+]([O-])(CCOc3cc(=O)oc4ccccc34)N=N2)c2ccccc21
**6a1**	18	0.270	*N*-dealkylation	O=CCOc1cc(=O)oc2ccccc12
**6a1**	18	0.270	*N*-dealkylation	O=C1C(=O)N(Cc2c[nH]nn2)c2ccccc21
**6a1**	18	0.270	aliphatic hydroxylation	O=C1C(=O)N(Cc2cn(C(O)COc3cc(=O)oc4ccccc34)nn2)c2ccccc21
**6a1**	22	0.191	alkyl dehydrogenation	O=C1C(=O)N(Cc2cn(C=COc3cc(=O)oc4ccccc34)nn2)c2ccccc21

Compound	Rank	Priority score	Predicted transformation	Predicted metabolite SMILES
**6a1**	23	0.140	aromatic hydroxylation	O=C1C(=O)N(Cc2nnn(CCOc3cc(=O)oc4ccccc34)c2O)c2ccccc21
**6a1**	23	0.140	dehydration of N-C bond	O=C1C(=O)[N+](=Cc2cn(CCOc3cc(=O)oc4ccccc34)nn2)c2ccccc21
**6a1**	23	0.140	epoxidation	O=C1C(=O)N(Cc2cn(CCOC34OC3C(=O)Oc3ccccc34)nn2)c2ccccc21
**6a2 — Parent SMILES:** O=C1C(=O)N(Cc2cn(CCOC3=CC(=O)Oc4ccccc34)nn2)c2ccc(F)cc21				
**6a2**	1	2.730	aromatic hydroxylation	O=C1C(=O)N(Cc2cn(CCOc3cc(=O)oc4cc(O)ccc34)nn2)c2ccc(F)cc21
**6a2**	2	1.610	aromatic hydroxylation	O=C1C(=O)N(Cc2cn(CCOc3cc(=O)oc4ccc(O)cc34)nn2)c2ccc(F)cc21
**6a2**	3	1.309	aromatic hydroxylation	O=C1C(=O)N(Cc2cn(CCOc3cc(=O)oc4c(O)cccc34)nn2)c2ccc(F)cc21
**6a2**	4	1.090	aromatic hydroxylation	O=C1C(=O)N(Cc2cn(CCOc3cc(=O)oc4ccccc34)nn2)c2c(O)cc(F)cc21
**6a2**	5	0.955	oxidative ether cleavage to one alcohol and one aldehyde/ketone	O=CCn1cc(CN2C(=O)C(=O)c3cc(F)ccc32)nn1
**6a2**	5	0.955	oxidative ether cleavage to one alcohol and one aldehyde/ketone	O=c1cc(O)c2ccccc2o1
**6a2**	5	0.955	aliphatic hydroxylation	O=C1C(=O)N(Cc2cn(CC(O)Oc3cc(=O)oc4ccccc34)nn2)c2ccc(F)cc21
**6a2**	8	0.860	aromatic hydroxylation	O=C1C(=O)N(Cc2cn(CCOc3cc(=O)oc4cccc(O)c34)nn2)c2ccc(F)cc21
**6a2**	9	0.850	aromatic hydroxylation	O=C1C(=O)N(Cc2cn(CCOc3cc(=O)oc4ccccc34)nn2)c2cc(O)c(F)cc21
**6a2**	10	0.700	*N*-dealkylation	O=Cc1cn(CCOc2cc(=O)oc3ccccc23)nn1
**6a2**	10	0.700	*N*-dealkylation	O=C1Nc2ccc(F)cc2C1=O
**6a2**	10	0.700	*N*-oxidation	O=C1C(=O)[N+]([O-])(Cc2cn(CCOc3cc(=O)oc4ccccc34)nn2)c2ccc(F)cc21
**6a2**	10	0.700	aliphatic hydroxylation	O=C1C(=O)N(C(O)c2cn(CCOc3cc(=O)oc4ccccc34)nn2)c2ccc(F)cc21
**6a2**	14	0.630	aromatic hydroxylation	O=C1C(=O)N(Cc2cn(CCOc3cc(=O)oc4ccccc34)nn2)c2ccc(F)c(O)c21
**6a2**	15	0.546	oxidation of 4-substituted anisole to quinone	O=CC=C(OCCn1cc(CN2C(=O)C(=O)c3cc(F)ccc32)nn1)C1=CC(=O)C=CC1=O
**6a2**	16	0.460	*N*-oxidation	O=C1C(=O)N(Cc2cn(CCOc3cc(=O)oc4ccccc34)n[n+]2[O-])c2ccc(F)cc21
**6a2**	17	0.270	*N*-oxidation	O=C1C(=O)N(CC2=C[N+]([O-])(CCOc3cc(=O)oc4ccccc34)N=N2)c2ccc(F)cc21
**6a2**	17	0.270	*N*-dealkylation	O=CCOc1cc(=O)oc2ccccc12
**6a2**	17	0.270	*N*-dealkylation	O=C1C(=O)N(Cc2c[nH]nn2)c2ccc(F)cc21
**6a2**	17	0.270	aliphatic hydroxylation	O=C1C(=O)N(Cc2cn(C(O)COc3cc(=O)oc4ccccc34)nn2)c2ccc(F)cc21
**6a2**	21	0.191	alkyl dehydrogenation	O=C1C(=O)N(Cc2cn(C=COc3cc(=O)oc4ccccc34)nn2)c2ccc(F)cc21

Compound	Rank	Priority score	Predicted transformation	Predicted metabolite SMILES
**6a2**	22	0.140	dehydration of N-C bond	O=C1C(=O)[N+](=Cc2cn(CCOc3cc(=O)oc4ccccc34)nn2)c2ccc(F)cc21
**6a2**	22	0.140	epoxidation	O=C1C(=O)N(Cc2cn(CCOC34OC3C(=O)Oc3ccccc34)nn2)c2ccc(F)cc21
**6a2**	22	0.140	aromatic hydroxylation	O=C1C(=O)N(Cc2nnn(CCOc3cc(=O)oc4ccccc34)c2O)c2ccc(F)cc21
**6a2**	25	0.084	oxidative dehalogenation benzyl	O=C1C(=O)N(Cc2cn(CCOc3cc(=O)oc4ccccc34)nn2)c2ccc(O)cc21
**6a3 — Parent SMILES:** O=C1C(=O)N(Cc2cn(CCOC3=CC(=O)Oc4ccccc34)nn2)c2ccc(Cl)cc21				
**6a3**	1	2.730	aromatic hydroxylation	O=C1C(=O)N(Cc2cn(CCOc3cc(=O)oc4cc(O)ccc34)nn2)c2ccc(Cl)cc21
**6a3**	2	1.610	aromatic hydroxylation	O=C1C(=O)N(Cc2cn(CCOc3cc(=O)oc4ccc(O)cc34)nn2)c2ccc(Cl)cc21
**6a3**	3	1.309	aromatic hydroxylation	O=C1C(=O)N(Cc2cn(CCOc3cc(=O)oc4c(O)cccc34)nn2)c2ccc(Cl)cc21
**6a3**	4	0.955	oxidative ether cleavage to one alcohol and one aldehyde/ketone	O=c1cc(O)c2ccccc2o1
**6a3**	4	0.955	oxidative ether cleavage to one alcohol and one aldehyde/ketone	O=CCn1cc(CN2C(=O)C(=O)c3cc(Cl)ccc32)nn1
**6a3**	4	0.955	aliphatic hydroxylation	O=C1C(=O)N(Cc2cn(CC(O)Oc3cc(=O)oc4ccccc34)nn2)c2ccc(Cl)cc21
**6a3**	7	0.950	aromatic hydroxylation	O=C1C(=O)N(Cc2cn(CCOc3cc(=O)oc4ccccc34)nn2)c2c(O)cc(Cl)cc21
**6a3**	8	0.860	aromatic hydroxylation	O=C1C(=O)N(Cc2cn(CCOc3cc(=O)oc4cccc(O)c34)nn2)c2ccc(Cl)cc21
**6a3**	9	0.700	*N*-dealkylation	O=Cc1cn(CCOc2cc(=O)oc3ccccc23)nn1
**6a3**	9	0.700	aliphatic hydroxylation	O=C1C(=O)N(C(O)c2cn(CCOc3cc(=O)oc4ccccc34)nn2)c2ccc(Cl)cc21
**6a3**	9	0.700	*N*-oxidation	O=C1C(=O)[N+]([O-])(Cc2cn(CCOc3cc(=O)oc4ccccc34)nn2)c2ccc(Cl)cc21
**6a3**	9	0.700	*N*-dealkylation	O=C1Nc2ccc(Cl)cc2C1=O
**6a3**	13	0.620	aromatic hydroxylation	O=C1C(=O)N(Cc2cn(CCOc3cc(=O)oc4ccccc34)nn2)c2ccc(Cl)c(O)c21
**6a3**	14	0.546	oxidation of 4-substituted anisole to quinone	O=CC=C(OCCn1cc(CN2C(=O)C(=O)c3cc(Cl)ccc32)nn1)C1=CC(=O)C=CC1=O
**6a3**	15	0.540	aromatic hydroxylation	O=C1C(=O)N(Cc2cn(CCOc3cc(=O)oc4ccccc34)nn2)c2cc(O)c(Cl)cc21
**6a3**	16	0.460	*N*-oxidation	O=C1C(=O)N(Cc2cn(CCOc3cc(=O)oc4ccccc34)n[n+]2[O-])c2ccc(Cl)cc21
**6a3**	17	0.270	aliphatic hydroxylation	O=C1C(=O)N(Cc2cn(C(O)COc3cc(=O)oc4ccccc34)nn2)c2ccc(Cl)cc21
**6a3**	17	0.270	*N*-oxidation	O=C1C(=O)N(CC2=C[N+]([O-])(CCOc3cc(=O)oc4ccccc34)N=N2)c2ccc(Cl)cc21
**6a3**	17	0.270	*N*-dealkylation	O=CCOc1cc(=O)oc2ccccc12
**6a3**	17	0.270	*N*-dealkylation	O=C1C(=O)N(Cc2c[nH]nn2)c2ccc(Cl)cc21

Compound	Rank	Priority score	Predicted transformation	Predicted metabolite SMILES
**6a3**	21	0.191	alkyl dehydrogenation	O=C1C(=O)N(Cc2cn(C=COc3cc(=O)oc4ccccc34)nn2)c2ccc(Cl)cc21
**6a3**	22	0.140	dehydration of N-C bond	O=C1C(=O)[N+](=Cc2cn(CCOc3cc(=O)oc4ccccc34)nn2)c2ccc(Cl)cc21
**6a3**	22	0.140	epoxidation	O=C1C(=O)N(Cc2cn(CCOC34OC3C(=O)Oc3ccccc34)nn2)c2ccc(Cl)cc21
**6a3**	24	0.130	aromatic hydroxylation	O=C1C(=O)N(Cc2nnn(CCOc3cc(=O)oc4ccccc34)c2O)c2ccc(Cl)cc21
**6a3**	25	0.040	oxidative dehalogenation benzyl	O=C1C(=O)N(Cc2cn(CCOc3cc(=O)oc4ccccc34)nn2)c2ccc(O)cc21
**6a4 — Parent SMILES:** O=C1C(=O)N(Cc2cn(CCOC3=CC(=O)Oc4ccccc34)nn2)c2ccc(Br)cc21				
**6a4**	1	2.730	aromatic hydroxylation	O=C1C(=O)N(Cc2cn(CCOc3cc(=O)oc4cc(O)ccc34)nn2)c2ccc(Br)cc21
**6a4**	2	1.610	aromatic hydroxylation	O=C1C(=O)N(Cc2cn(CCOc3cc(=O)oc4ccc(O)cc34)nn2)c2ccc(Br)cc21
**6a4**	3	1.309	aromatic hydroxylation	O=C1C(=O)N(Cc2cn(CCOc3cc(=O)oc4c(O)cccc34)nn2)c2ccc(Br)cc21
**6a4**	4	0.955	oxidative ether cleavage to one alcohol and one aldehyde/ketone	O=c1cc(O)c2ccccc2o1
**6a4**	4	0.955	aliphatic hydroxylation	O=C1C(=O)N(Cc2cn(CC(O)Oc3cc(=O)oc4ccccc34)nn2)c2ccc(Br)cc21
**6a4**	4	0.955	oxidative ether cleavage to one alcohol and one aldehyde/ketone	O=CCn1cc(CN2C(=O)C(=O)c3cc(Br)ccc32)nn1
**6a4**	7	0.940	aromatic hydroxylation	O=C1C(=O)N(Cc2cn(CCOc3cc(=O)oc4ccccc34)nn2)c2c(O)cc(Br)cc21
**6a4**	8	0.860	aromatic hydroxylation	O=C1C(=O)N(Cc2cn(CCOc3cc(=O)oc4cccc(O)c34)nn2)c2ccc(Br)cc21
**6a4**	9	0.690	*N*-dealkylation	O=Cc1cn(CCOc2cc(=O)oc3ccccc23)nn1
**6a4**	9	0.690	aliphatic hydroxylation	O=C1C(=O)N(C(O)c2cn(CCOc3cc(=O)oc4ccccc34)nn2)c2ccc(Br)cc21
**6a4**	9	0.690	*N*-oxidation	O=C1C(=O)[N+]([O-])(Cc2cn(CCOc3cc(=O)oc4ccccc34)nn2)c2ccc(Br)cc21
**6a4**	9	0.690	*N*-dealkylation	O=C1Nc2ccc(Br)cc2C1=O
**6a4**	13	0.590	aromatic hydroxylation	O=C1C(=O)N(Cc2cn(CCOc3cc(=O)oc4ccccc34)nn2)c2ccc(Br)c(O)c21
**6a4**	14	0.546	oxidation of 4-substituted anisole to quinone	O=CC=C(OCCn1cc(CN2C(=O)C(=O)c3cc(Br)ccc32)nn1)C1=CC(=O)C=CC1=O
**6a4**	15	0.460	*N*-oxidation	O=C1C(=O)N(Cc2cn(CCOc3cc(=O)oc4ccccc34)n[n+]2[O-])c2ccc(Br)cc21
**6a4**	15	0.460	aromatic hydroxylation	O=C1C(=O)N(Cc2cn(CCOc3cc(=O)oc4ccccc34)nn2)c2cc(O)c(Br)cc21
**6a4**	17	0.270	*N*-oxidation	O=C1C(=O)N(CC2=C[N+]([O-])(CCOc3cc(=O)oc4ccccc34)N=N2)c2ccc(Br)cc21
**6a4**	17	0.270	*N*-dealkylation	O=C1C(=O)N(Cc2c[nH]nn2)c2ccc(Br)cc21
**6a4**	17	0.270	*N*-dealkylation	O=CCOc1cc(=O)oc2ccccc12

Compound	Rank	Priority score	Predicted transformation	Predicted metabolite SMILES
**6a4**	17	0.270	aliphatic hydroxylation	O=C1C(=O)N(Cc2cn(C(O)COc3cc(=O)oc4ccccc34)nn2)c2ccc(Br)cc21
**6a4**	21	0.191	alkyl dehydrogenation	O=C1C(=O)N(Cc2cn(C=COc3cc(=O)oc4ccccc34)nn2)c2ccc(Br)cc21
**6a4**	22	0.140	epoxidation	O=C1C(=O)N(Cc2cn(CCOC34OC3C(=O)Oc3ccccc34)nn2)c2ccc(Br)cc21
**6a4**	23	0.138	dehydration of N-C bond	O=C1C(=O)[N+](=Cc2cn(CCOc3cc(=O)oc4ccccc34)nn2)c2ccc(Br)cc21
**6a4**	24	0.130	aromatic hydroxylation	O=C1C(=O)N(Cc2nnn(CCOc3cc(=O)oc4ccccc34)c2O)c2ccc(Br)cc21
**6a4**	25	0.098	oxidative dehalogenation benzyl	O=C1C(=O)N(Cc2cn(CCOc3cc(=O)oc4ccccc34)nn2)c2ccc(O)cc21
**6a5 — Parent SMILES:** O=C1C(=O)N(Cc2cn(CCOC3=CC(=O)Oc4ccccc34)nn2)c2ccc(I)cc21				
**6a5**	1	2.730	aromatic hydroxylation	O=C1C(=O)N(Cc2cn(CCOc3cc(=O)oc4cc(O)ccc34)nn2)c2ccc(I)cc21
**6a5**	2	1.610	aromatic hydroxylation	O=C1C(=O)N(Cc2cn(CCOc3cc(=O)oc4ccc(O)cc34)nn2)c2ccc(I)cc21
**6a5**	3	1.309	aromatic hydroxylation	O=C1C(=O)N(Cc2cn(CCOc3cc(=O)oc4c(O)cccc34)nn2)c2ccc(I)cc21
**6a5**	4	0.955	oxidative ether cleavage to one alcohol and one aldehyde/ketone	O=c1cc(O)c2ccccc2o1
**6a5**	4	0.955	oxidative ether cleavage to one alcohol and one aldehyde/ketone	O=CCn1cc(CN2C(=O)C(=O)c3cc(I)ccc32)nn1
**6a5**	4	0.955	aliphatic hydroxylation	O=C1C(=O)N(Cc2cn(CC(O)Oc3cc(=O)oc4ccccc34)nn2)c2ccc(I)cc21
**6a5**	7	0.900	aromatic hydroxylation	O=C1C(=O)N(Cc2cn(CCOc3cc(=O)oc4ccccc34)nn2)c2c(O)cc(I)cc21
**6a5**	8	0.860	aromatic hydroxylation	O=C1C(=O)N(Cc2cn(CCOc3cc(=O)oc4cccc(O)c34)nn2)c2ccc(I)cc21
**6a5**	9	0.690	aliphatic hydroxylation	O=C1C(=O)N(C(O)c2cn(CCOc3cc(=O)oc4ccccc34)nn2)c2ccc(I)cc21
**6a5**	9	0.690	*N*-dealkylation	O=Cc1cn(CCOc2cc(=O)oc3ccccc23)nn1
**6a5**	9	0.690	*N*-dealkylation	O=C1Nc2ccc(I)cc2C1=O
**6a5**	9	0.690	*N*-oxidation	O=C1C(=O)[N+]([O-])(Cc2cn(CCOc3cc(=O)oc4ccccc34)nn2)c2ccc(I)cc21
**6a5**	13	0.546	oxidation of 4-substituted anisole to quinone	O=CC=C(OCCn1cc(CN2C(=O)C(=O)c3cc(I)ccc32)nn1)C1=CC(=O)C=CC1=O
**6a5**	14	0.530	aromatic hydroxylation	O=C1C(=O)N(Cc2cn(CCOc3cc(=O)oc4ccccc34)nn2)c2ccc(I)c(O)c21
**6a5**	15	0.480	aromatic hydroxylation	O=C1C(=O)N(Cc2cn(CCOc3cc(=O)oc4ccccc34)nn2)c2cc(O)c(I)cc21
**6a5**	16	0.450	*N*-oxidation	O=C1C(=O)N(Cc2cn(CCOc3cc(=O)oc4ccccc34)n[n+]2[O-])c2ccc(I)cc21
**6a5**	17	0.270	aliphatic hydroxylation	O=C1C(=O)N(Cc2cn(C(O)COc3cc(=O)oc4ccccc34)nn2)c2ccc(I)cc21
**6a5**	17	0.270	*N*-oxidation	O=C1C(=O)N(CC2=C[N+]([O-])(CCOc3cc(=O)oc4ccccc34)N=N2)c2ccc(I)cc21

Compound	Rank	Priority score	Predicted transformation	Predicted metabolite SMILES
**6a5**	17	0.270	*N*-dealkylation	O=CCOc1cc(=O)oc2ccccc12
**6a5**	17	0.270	*N*-dealkylation	O=C1C(=O)N(Cc2c[nH]nn2)c2ccc(I)cc21
**6a5**	21	0.236	oxidative dehalogenation benzyl	O=C1C(=O)N(Cc2cn(CCOc3cc(=O)oc4ccccc34)nn2)c2ccc(O)cc21
**6a5**	22	0.191	alkyl dehydrogenation	O=C1C(=O)N(Cc2cn(C=COc3cc(=O)oc4ccccc34)nn2)c2ccc(I)cc21
**6a5**	23	0.140	epoxidation	O=C1C(=O)N(Cc2cn(CCOC34OC3C(=O)Oc3ccccc34)nn2)c2ccc(I)cc21
**6a5**	24	0.138	dehydration of N-C bond	O=C1C(=O)[N+](=Cc2cn(CCOc3cc(=O)oc4ccccc34)nn2)c2ccc(I)cc21
**6a5**	25	0.120	aromatic hydroxylation	O=C1C(=O)N(Cc2nnn(CCOc3cc(=O)oc4ccccc34)c2O)c2ccc(I)cc21
**6a6 — Parent SMILES:** O=C1C(=O)N(Cc2cn(CCOC3=CC(=O)Oc4ccccc34)nn2)c2ccc([N+](=O)[O-])cc21				
**6a6**	1	2.650	aromatic hydroxylation	O=C1C(=O)N(Cc2cn(CCOc3cc(=O)oc4cc(O)ccc34)nn2)c2ccc([N+](=O)[O-])cc21
**6a6**	2	1.590	aromatic hydroxylation	O=C1C(=O)N(Cc2cn(CCOc3cc(=O)oc4ccc(O)cc34)nn2)c2ccc([N+](=O)[O-])cc21
**6a6**	3	1.329	aromatic hydroxylation	O=C1C(=O)N(Cc2cn(CCOc3cc(=O)oc4c(O)cccc34)nn2)c2ccc([N+](=O)[O-])cc21
**6a6**	4	0.915	aliphatic hydroxylation	O=C1C(=O)N(Cc2cn(CC(O)Oc3cc(=O)oc4ccccc34)nn2)c2ccc([N+](=O)[O-])cc21
**6a6**	4	0.915	oxidative ether cleavage to one alcohol and one aldehyde/ketone	O=CCn1cc(CN2C(=O)C(=O)c3cc([N+](=O)[O-])ccc32)nn1
**6a6**	4	0.915	oxidative ether cleavage to one alcohol and one aldehyde/ketone	O=c1cc(O)c2ccccc2o1
**6a6**	7	0.830	aromatic hydroxylation	O=C1C(=O)N(Cc2cn(CCOc3cc(=O)oc4cccc(O)c34)nn2)c2ccc([N+](=O)[O-])cc21
**6a6**	8	0.800	aromatic hydroxylation	O=C1C(=O)N(Cc2cn(CCOc3cc(=O)oc4ccccc34)nn2)c2cc(O)c([N+](=O)[O-])cc21
**6a6**	9	0.770	aromatic hydroxylation	O=C1C(=O)N(Cc2cn(CCOc3cc(=O)oc4ccccc34)nn2)c2ccc([N+](=O)[O-])c(O)c21
**6a6**	10	0.640	*N*-oxidation	O=C1C(=O)[N+]([O-])(Cc2cn(CCOc3cc(=O)oc4ccccc34)nn2)c2ccc([N+](=O)[O-])cc21
**6a6**	10	0.640	*N*-dealkylation	O=Cc1cn(CCOc2cc(=O)oc3ccccc23)nn1
**6a6**	10	0.640	*N*-dealkylation	O=C1Nc2ccc([N+](=O)[O-])cc2C1=O
**6a6**	10	0.640	aliphatic hydroxylation	O=C1C(=O)N(C(O)c2cn(CCOc3cc(=O)oc4ccccc34)nn2)c2ccc([N+](=O)[O-])cc21
**6a6**	14	0.617	aromatic hydroxylation	O=C1C(=O)N(Cc2cn(CCOc3cc(=O)oc4ccccc34)nn2)c2c(O)cc([N+](=O)[O-])cc21
**6a6**	16	0.530	oxidation of 4-substituted anisole to quinone	O=CC=C(OCCn1cc(CN2C(=O)C(=O)c3cc([N+](=O)[O-])ccc32)nn1)C1=CC(=O)C=CC1=O
**6a6**	17	0.440	*N*-oxidation	O=C1C(=O)N(Cc2cn(CCOc3cc(=O)oc4ccccc34)n[n+]2[O-])c2ccc([N+](=O)[O-])cc21
**6a6**	18	0.260	*N*-dealkylation	O=C1C(=O)N(Cc2c[nH]nn2)c2ccc([N+](=O)[O-])cc21

Compound	Rank	Priority score	Predicted transformation	Predicted metabolite SMILES
**6a6**	18	0.260	*N*-dealkylation	O=CCOc1cc(=O)oc2ccccc12
**6a6**	18	0.260	*N*-oxidation	O=C1C(=O)N(CC2=C[N+]([O-])(CCOc3cc(=O)oc4ccccc34)N=N2)c2ccc([N+](=O)[O-])cc21
**6a6**	18	0.260	aliphatic hydroxylation	O=C1C(=O)N(Cc2cn(C(O)COc3cc(=O)oc4ccccc34)nn2)c2ccc([N+](=O)[O-])cc21
**6a6**	22	0.183	alkyl dehydrogenation	O=C1C(=O)N(Cc2cn(C=COc3cc(=O)oc4ccccc34)nn2)c2ccc([N+](=O)[O-])cc21
**6a6**	23	0.180	aromatic hydroxylation	O=C1C(=O)N(Cc2nnn(CCOc3cc(=O)oc4ccccc34)c2O)c2ccc([N+](=O)[O-])cc21
**6a6**	24	0.150	epoxidation	O=C1C(=O)N(Cc2cn(CCOC34OC3C(=O)Oc3ccccc34)nn2)c2ccc([N+](=O)[O-])cc21
**6a6**	25	0.128	dehydration of N-C bond	O=C1C(=O)[N+](=Cc2cn(CCOc3cc(=O)oc4ccccc34)nn2)c2ccc([N+](=O)[O-])cc21
**6a6**	26	0.112	reduction of nitro group	Nc1ccc2c(c1)C(=O)C(=O)N2Cc1cn(CCOc2cc(=O)oc3ccccc23)nn1
**6a7 — Parent SMILES:** O=C1C(=O)N(Cc2cn(CCOC3=CC(=O)Oc4ccccc34)nn2)c2ccc(C)cc21				
**6a7**	1	3.490	aliphatic hydroxylation	O=C1C(=O)N(Cc2cn(CCOc3cc(=O)oc4ccccc34)nn2)c2ccc(CO)cc21
**6a7**	2	2.650	aromatic hydroxylation	Cc1ccc2c(c1)C(=O)C(=O)N2Cc1cn(CCOc2cc(=O)oc3cc(O)ccc23)nn1
**6a7**	3	1.590	aromatic hydroxylation	Cc1ccc2c(c1)C(=O)C(=O)N2Cc1cn(CCOc2cc(=O)oc3ccc(O)cc23)nn1
**6a7**	4	1.329	aromatic hydroxylation	Cc1ccc2c(c1)C(=O)C(=O)N2Cc1cn(CCOc2cc(=O)oc3c(O)cccc23)nn1
**6a7**	5	0.915	aliphatic hydroxylation	Cc1ccc2c(c1)C(=O)C(=O)N2Cc1cn(CC(O)Oc2cc(=O)oc3ccccc23)nn1
**6a7**	5	0.915	oxidative ether cleavage to one alcohol and one aldehyde/ketone	Cc1ccc2c(c1)C(=O)C(=O)N2Cc1cn(CC=O)nn1
**6a7**	5	0.915	oxidative ether cleavage to one alcohol and one aldehyde/ketone	O=c1cc(O)c2ccccc2o1
**6a7**	8	0.830	aromatic hydroxylation	Cc1ccc2c(c1)C(=O)C(=O)N2Cc1cn(CCOc2cc(=O)oc3cccc(O)c23)nn1
**6a7**	9	0.660	aromatic hydroxylation	Cc1cc2c(cc1O)N(Cc1cn(CCOc3cc(=O)oc4ccccc34)nn1)C(=O)C2=O
**6a7**	10	0.590	aromatic hydroxylation	Cc1cc(O)c2c(c1)C(=O)C(=O)N2Cc1cn(CCOc2cc(=O)oc3ccccc23)nn1
**6a7**	11	0.570	aromatic hydroxylation	Cc1ccc2c(c1O)C(=O)C(=O)N2Cc1cn(CCOc2cc(=O)oc3ccccc23)nn1
**6a7**	12	0.540	*N*-dealkylation	Cc1ccc2c(c1)C(=O)C(=O)N2
**6a7**	12	0.540	*N*-dealkylation	O=Cc1cn(CCOc2cc(=O)oc3ccccc23)nn1
**6a7**	12	0.540	aliphatic hydroxylation	Cc1ccc2c(c1)C(=O)C(=O)N2C(O)c1cn(CCOc2cc(=O)oc3ccccc23)nn1
**6a7**	12	0.540	*N*-oxidation	Cc1ccc2c(c1)C(=O)C(=O)[N+]2([O-])Cc1cn(CCOc2cc(=O)oc3ccccc23)nn1

Compound	Rank	Priority score	Predicted transformation	Predicted metabolite SMILES
**6a7**	16	0.530	oxidation of 4-substituted anisole to quinone	Cc1ccc2c(c1)C(=O)C(=O)N2Cc1cn(CCOC(=CC=O)C2=CC(=O)C=CC2=O)nn1
**6a7**	17	0.440	*N*-oxidation	Cc1ccc2c(c1)C(=O)C(=O)N2Cc1cn(CCOc2cc(=O)oc3ccccc23)n[n+]1[O-]
**6a7**	18	0.260	*N*-oxidation	Cc1ccc2c(c1)C(=O)C(=O)N2CC1=C[N+]([O-])(CCOc2cc(=O)oc3ccccc23)N=N1
**6a7**	18	0.260	*N*-dealkylation	O=CCOc1cc(=O)oc2ccccc12
**6a7**	18	0.260	aliphatic hydroxylation	Cc1ccc2c(c1)C(=O)C(=O)N2Cc1cn(C(O)COc2cc(=O)oc3ccccc23)nn1
**6a7**	18	0.260	*N*-dealkylation	Cc1ccc2c(c1)C(=O)C(=O)N2Cc1c[nH]nn1
**6a7**	22	0.183	alkyl dehydrogenation	Cc1ccc2c(c1)C(=O)C(=O)N2Cc1cn(C=COc2cc(=O)oc3ccccc23)nn1
**6a7**	23	0.180	aromatic hydroxylation	Cc1ccc2c(c1)C(=O)C(=O)N2Cc1nnn(CCOc2cc(=O)oc3ccccc23)c1O
**6a7**	24	0.150	epoxidation	Cc1ccc2c(c1)C(=O)C(=O)N2Cc1cn(CCOC23OC2C(=O)Oc2ccccc23)nn1
**6a7**	25	0.108	dehydration of N-C bond	Cc1ccc2c(c1)C(=O)C(=O)[N+]2=Cc1cn(CCOc2cc(=O)oc3ccccc23)nn1
**Series 6b**				
**6b1 — Parent SMILES:** O=C1C(=O)N(Cc2cn(CCCOC3=CC(=O)Oc4ccccc34)nn2)c2ccccc21				
**6b1**	1	2.700	aromatic hydroxylation	O=C1C(=O)N(Cc2cn(CCCOc3cc(=O)oc4cc(O)ccc34)nn2)c2ccccc21
**6b1**	2	2.480	aromatic hydroxylation	O=C1C(=O)N(Cc2cn(CCCOc3cc(=O)oc4ccccc34)nn2)c2ccc(O)cc21
**6b1**	3	1.660	aromatic hydroxylation	O=C1C(=O)N(Cc2cn(CCCOc3cc(=O)oc4ccc(O)cc34)nn2)c2ccccc21
**6b1**	4	1.325	aromatic hydroxylation	O=C1C(=O)N(Cc2cn(CCCOc3cc(=O)oc4c(O)cccc34)nn2)c2ccccc21
**6b1**	5	1.140	aromatic hydroxylation	O=C1C(=O)N(Cc2cn(CCCOc3cc(=O)oc4ccccc34)nn2)c2c(O)cccc21
**6b1**	6	0.920	oxidative ether cleavage to one alcohol and one aldehyde/ketone	O=c1cc(O)c2ccccc2o1
**6b1**	6	0.920	oxidative ether cleavage to one alcohol and one aldehyde/ketone	O=CCCn1cc(CN2C(=O)C(=O)c3ccccc32)nn1
**6b1**	6	0.920	aliphatic hydroxylation	O=C1C(=O)N(Cc2cn(CCC(O)Oc3cc(=O)oc4ccccc34)nn2)c2ccccc21
**6b1**	9	0.830	aromatic hydroxylation	O=C1C(=O)N(Cc2cn(CCCOc3cc(=O)oc4ccccc34)nn2)c2cc(O)ccc21
**6b1**	10	0.700	aromatic hydroxylation	O=C1C(=O)N(Cc2cn(CCCOc3cc(=O)oc4ccccc34)nn2)c2cccc(O)c21
**6b1**	11	0.670	aromatic hydroxylation	O=C1C(=O)N(Cc2cn(CCCOc3cc(=O)oc4cccc(O)c34)nn2)c2ccccc21
**6b1**	12	0.650	aliphatic hydroxylation	O=C1C(=O)N(C(O)c2cn(CCCOc3cc(=O)oc4ccccc34)nn2)c2ccccc21
**6b1**	12	0.650	*N*-dealkylation	O=Cc1cn(CCCOc2cc(=O)oc3ccccc23)nn1

Compound	Rank	Priority score	Predicted transformation	Predicted metabolite SMILES
**6b1**	12	0.650	*N*-oxidation	O=C1C(=O)[N+]([O-])(Cc2cn(CCCOc3cc(=O)oc4ccccc34)nn2)c2ccccc21
**6b1**	12	0.650	*N*-dealkylation	O=C1Nc2ccccc2C1=O
**6b1**	16	0.540	oxidation of 4-substituted anisole to quinone	O=CC=C(OCCCn1cc(CN2C(=O)C(=O)c3ccccc32)nn1)C1=CC(=O)C=CC1=O
**6b1**	17	0.410	aliphatic hydroxylation	O=C1C(=O)N(Cc2cn(CC(O)COc3cc(=O)oc4ccccc34)nn2)c2ccccc21
**6b1**	18	0.340	aliphatic hydroxylation	O=C1C(=O)N(Cc2cn(C(O)CCOc3cc(=O)oc4ccccc34)nn2)c2ccccc21
**6b1**	18	0.340	*N*-oxidation	O=C1C(=O)N(CC2=C[N+]([O-])(CCCOc3cc(=O)oc4ccccc34)N=N2)c2ccccc21
**6b1**	18	0.340	*N*-dealkylation	O=CCCOc1cc(=O)oc2ccccc12
**6b1**	18	0.340	*N*-dealkylation	O=C1C(=O)N(Cc2c[nH]nn2)c2ccccc21
**6b1**	22	0.310	*N*-oxidation	O=C1C(=O)N(Cc2cn(CCCOc3cc(=O)oc4ccccc34)n[n+]2[O-])c2ccccc21
**6b1**	23	0.184	alkyl dehydrogenation	O=C1C(=O)N(Cc2cn(CC=COc3cc(=O)oc4ccccc34)nn2)c2ccccc21
**6b1**	24	0.160	epoxidation	O=C1C(=O)N(Cc2cn(CCCOC34OC3C(=O)Oc3ccccc34)nn2)c2ccccc21
**6b1**	25	0.150	aromatic hydroxylation	O=C1C(=O)N(Cc2nnn(CCCOc3cc(=O)oc4ccccc34)c2O)c2ccccc21
**6b1**	26	0.130	dehydration of N-C bond	O=C1C(=O)[N+](=Cc2cn(CCCOc3cc(=O)oc4ccccc34)nn2)c2ccccc21
**6b1**	27	0.082	alkyl dehydrogenation	O=C1C(=O)N(Cc2cn(C=CCOc3cc(=O)oc4ccccc34)nn2)c2ccccc21
**6b2 — Parent SMILES:** O=C1C(=O)N(Cc2cn(CCCOC3=CC(=O)Oc4ccccc34)nn2)c2ccc(F)cc21				
**6b2**	1	2.700	aromatic hydroxylation	O=C1C(=O)N(Cc2cn(CCCOc3cc(=O)oc4cc(O)ccc34)nn2)c2ccc(F)cc21
**6b2**	2	1.660	aromatic hydroxylation	O=C1C(=O)N(Cc2cn(CCCOc3cc(=O)oc4ccc(O)cc34)nn2)c2ccc(F)cc21
**6b2**	3	1.325	aromatic hydroxylation	O=C1C(=O)N(Cc2cn(CCCOc3cc(=O)oc4c(O)cccc34)nn2)c2ccc(F)cc21
**6b2**	4	1.090	aromatic hydroxylation	O=C1C(=O)N(Cc2cn(CCCOc3cc(=O)oc4ccccc34)nn2)c2c(O)cc(F)cc21
**6b2**	5	0.920	oxidative ether cleavage to one alcohol and one aldehyde/ketone	O=c1cc(O)c2ccccc2o1
**6b2**	5	0.920	aliphatic hydroxylation	O=C1C(=O)N(Cc2cn(CCC(O)Oc3cc(=O)oc4ccccc34)nn2)c2ccc(F)cc21
**6b2**	5	0.920	oxidative ether cleavage to one alcohol and one aldehyde/ketone	O=CCCn1cc(CN2C(=O)C(=O)c3cc(F)ccc32)nn1
**6b2**	8	0.900	aromatic hydroxylation	O=C1C(=O)N(Cc2cn(CCCOc3cc(=O)oc4ccccc34)nn2)c2cc(O)c(F)cc21
**6b2**	9	0.700	aromatic hydroxylation	O=C1C(=O)N(Cc2cn(CCCOc3cc(=O)oc4ccccc34)nn2)c2ccc(F)c(O)c21
**6b2**	10	0.670	aromatic hydroxylation	O=C1C(=O)N(Cc2cn(CCCOc3cc(=O)oc4cccc(O)c34)nn2)c2ccc(F)cc21

Compound	Rank	Priority score	Predicted transformation	Predicted metabolite SMILES
**6b2**	11	0.650	aliphatic hydroxylation	O=C1C(=O)N(C(O)c2cn(CCCOc3cc(=O)oc4ccccc34)nn2)c2ccc(F)cc21
**6b2**	11	0.650	*N*-dealkylation	O=C1Nc2ccc(F)cc2C1=O
**6b2**	11	0.650	*N*-dealkylation	O=Cc1cn(CCCOc2cc(=O)oc3ccccc23)nn1
**6b2**	11	0.650	*N*-oxidation	O=C1C(=O)[N+]([O-])(Cc2cn(CCCOc3cc(=O)oc4ccccc34)nn2)c2ccc(F)cc21
**6b2**	15	0.540	oxidation of 4-substituted anisole to quinone	O=CC=C(OCCCn1cc(CN2C(=O)C(=O)c3cc(F)ccc32)nn1)C1=CC(=O)C=CC1=O
**6b2**	16	0.410	aliphatic hydroxylation	O=C1C(=O)N(Cc2cn(CC(O)COc3cc(=O)oc4ccccc34)nn2)c2ccc(F)cc21
**6b2**	17	0.340	*N*-dealkylation	O=C1C(=O)N(Cc2c[nH]nn2)c2ccc(F)cc21
**6b2**	17	0.340	*N*-oxidation	O=C1C(=O)N(CC2=C[N+]([O-])(CCCOc3cc(=O)oc4ccccc34)N=N2)c2ccc(F)cc21
**6b2**	17	0.340	aliphatic hydroxylation	O=C1C(=O)N(Cc2cn(C(O)CCOc3cc(=O)oc4ccccc34)nn2)c2ccc(F)cc21
**6b2**	17	0.340	*N*-dealkylation	O=CCCOc1cc(=O)oc2ccccc12
**6b2**	21	0.310	*N*-oxidation	O=C1C(=O)N(Cc2cn(CCCOc3cc(=O)oc4ccccc34)n[n+]2[O-])c2ccc(F)cc21
**6b2**	22	0.184	alkyl dehydrogenation	O=C1C(=O)N(Cc2cn(CC=COc3cc(=O)oc4ccccc34)nn2)c2ccc(F)cc21
**6b2**	23	0.160	epoxidation	O=C1C(=O)N(Cc2cn(CCCOC34OC3C(=O)Oc3ccccc34)nn2)c2ccc(F)cc21
**6b2**	24	0.150	aromatic hydroxylation	O=C1C(=O)N(Cc2nnn(CCCOc3cc(=O)oc4ccccc34)c2O)c2ccc(F)cc21
**6b2**	25	0.130	dehydration of N-C bond	O=C1C(=O)[N+](=Cc2cn(CCCOc3cc(=O)oc4ccccc34)nn2)c2ccc(F)cc21
**6b2**	26	0.086	oxidative dehalogenation benzyl	O=C1C(=O)N(Cc2cn(CCCOc3cc(=O)oc4ccccc34)nn2)c2ccc(O)cc21
**6b2**	27	0.082	alkyl dehydrogenation	O=C1C(=O)N(Cc2cn(C=CCOc3cc(=O)oc4ccccc34)nn2)c2ccc(F)cc21
**6b3 — Parent SMILES:** O=C1C(=O)N(Cc2cn(CCCOC3=CC(=O)Oc4ccccc34)nn2)c2ccc(Cl)cc21				
**6b3**	1	2.700	aromatic hydroxylation	O=C1C(=O)N(Cc2cn(CCCOc3cc(=O)oc4cc(O)ccc34)nn2)c2ccc(Cl)cc21
**6b3**	2	1.660	aromatic hydroxylation	O=C1C(=O)N(Cc2cn(CCCOc3cc(=O)oc4ccc(O)cc34)nn2)c2ccc(Cl)cc21
**6b3**	3	1.325	aromatic hydroxylation	O=C1C(=O)N(Cc2cn(CCCOc3cc(=O)oc4c(O)cccc34)nn2)c2ccc(Cl)cc21
**6b3**	4	0.950	aromatic hydroxylation	O=C1C(=O)N(Cc2cn(CCCOc3cc(=O)oc4ccccc34)nn2)c2c(O)cc(Cl)cc21
**6b3**	5	0.920	aliphatic hydroxylation	O=C1C(=O)N(Cc2cn(CCC(O)Oc3cc(=O)oc4ccccc34)nn2)c2ccc(Cl)cc21
**6b3**	5	0.920	oxidative ether cleavage to one alcohol and one aldehyde/ketone	O=c1cc(O)c2ccccc2o1
**6b3**	5	0.920	oxidative ether cleavage to one alcohol and one aldehyde/ketone	O=CCCn1cc(CN2C(=O)C(=O)c3cc(Cl)ccc32)nn1

Compound	Rank	Priority score	Predicted transformation	Predicted metabolite SMILES
**6b3**	8	0.670	aromatic hydroxylation	O=C1C(=O)N(Cc2cn(CCCOc3cc(=O)oc4ccccc34)nn2)c2ccc(Cl)c(O)c21
**6b3**	9	0.660	aromatic hydroxylation	O=C1C(=O)N(Cc2cn(CCCOc3cc(=O)oc4cccc(O)c34)nn2)c2ccc(Cl)cc21
**6b3**	10	0.650	aliphatic hydroxylation	O=C1C(=O)N(C(O)c2cn(CCCOc3cc(=O)oc4ccccc34)nn2)c2ccc(Cl)cc21
**6b3**	10	0.650	*N*-oxidation	O=C1C(=O)[N+]([O-])(Cc2cn(CCCOc3cc(=O)oc4ccccc34)nn2)c2ccc(Cl)cc21
**6b3**	10	0.650	*N*-dealkylation	O=Cc1cn(CCCOc2cc(=O)oc3ccccc23)nn1
**6b3**	10	0.650	*N*-dealkylation	O=C1Nc2ccc(Cl)cc2C1=O
**6b3**	14	0.580	aromatic hydroxylation	O=C1C(=O)N(Cc2cn(CCCOc3cc(=O)oc4ccccc34)nn2)c2cc(O)c(Cl)cc21
**6b3**	15	0.540	oxidation of 4-substituted anisole to quinone	O=CC=C(OCCCn1cc(CN2C(=O)C(=O)c3cc(Cl)ccc32)nn1)C1=CC(=O)C=CC1=O
**6b3**	16	0.410	aliphatic hydroxylation	O=C1C(=O)N(Cc2cn(CC(O)COc3cc(=O)oc4ccccc34)nn2)c2ccc(Cl)cc21
**6b3**	17	0.340	aliphatic hydroxylation	O=C1C(=O)N(Cc2cn(C(O)CCOc3cc(=O)oc4ccccc34)nn2)c2ccc(Cl)cc21
**6b3**	17	0.340	*N*-oxidation	O=C1C(=O)N(CC2=C[N+]([O-])(CCCOc3cc(=O)oc4ccccc34)N=N2)c2ccc(Cl)cc21
**6b3**	17	0.340	*N*-dealkylation	O=CCCOc1cc(=O)oc2ccccc12
**6b3**	17	0.340	*N*-dealkylation	O=C1C(=O)N(Cc2c[nH]nn2)c2ccc(Cl)cc21
**6b3**	21	0.310	*N*-oxidation	O=C1C(=O)N(Cc2cn(CCCOc3cc(=O)oc4ccccc34)n[n+]2[O-])c2ccc(Cl)cc21
**6b3**	22	0.184	alkyl dehydrogenation	O=C1C(=O)N(Cc2cn(CC=COc3cc(=O)oc4ccccc34)nn2)c2ccc(Cl)cc21
**6b3**	23	0.160	epoxidation	O=C1C(=O)N(Cc2cn(CCCOC34OC3C(=O)Oc3ccccc34)nn2)c2ccc(Cl)cc21
**6b3**	24	0.150	aromatic hydroxylation	O=C1C(=O)N(Cc2nnn(CCCOc3cc(=O)oc4ccccc34)c2O)c2ccc(Cl)cc21
**6b3**	25	0.130	dehydration of N-C bond	O=C1C(=O)[N+](=Cc2cn(CCCOc3cc(=O)oc4ccccc34)nn2)c2ccc(Cl)cc21
**6b3**	26	0.082	alkyl dehydrogenation	O=C1C(=O)N(Cc2cn(C=CCOc3cc(=O)oc4ccccc34)nn2)c2ccc(Cl)cc21
**6b3**	27	0.042	oxidative dehalogenation benzyl	O=C1C(=O)N(Cc2cn(CCCOc3cc(=O)oc4ccccc34)nn2)c2ccc(O)cc21
**6b4 — Parent SMILES:** O=C1C(=O)N(Cc2cn(CCCOC3=CC(=O)Oc4ccccc34)nn2)c2ccc(Br)cc21				
**6b4**	1	2.700	aromatic hydroxylation	O=C1C(=O)N(Cc2cn(CCCOc3cc(=O)oc4cc(O)ccc34)nn2)c2ccc(Br)cc21
**6b4**	2	1.660	aromatic hydroxylation	O=C1C(=O)N(Cc2cn(CCCOc3cc(=O)oc4ccc(O)cc34)nn2)c2ccc(Br)cc21
**6b4**	3	1.325	aromatic hydroxylation	O=C1C(=O)N(Cc2cn(CCCOc3cc(=O)oc4c(O)cccc34)nn2)c2ccc(Br)cc21
**6b4**	4	0.940	aromatic hydroxylation	O=C1C(=O)N(Cc2cn(CCCOc3cc(=O)oc4ccccc34)nn2)c2c(O)cc(Br)cc21

Compound	Rank	Priority score	Predicted transformation	Predicted metabolite SMILES
**6b4**	5	0.920	oxidative ether cleavage to one alcohol and one aldehyde/ketone	O=c1cc(O)c2ccccc2o1
**6b4**	5	0.920	oxidative ether cleavage to one alcohol and one aldehyde/ketone	O=CCCn1cc(CN2C(=O)C(=O)c3cc(Br)ccc32)nn1
**6b4**	5	0.920	aliphatic hydroxylation	O=C1C(=O)N(Cc2cn(CCC(O)Oc3cc(=O)oc4ccccc34)nn2)c2ccc(Br)cc21
**6b4**	8	0.660	aromatic hydroxylation	O=C1C(=O)N(Cc2cn(CCCOc3cc(=O)oc4cccc(O)c34)nn2)c2ccc(Br)cc21
**6b4**	9	0.650	aromatic hydroxylation	O=C1C(=O)N(Cc2cn(CCCOc3cc(=O)oc4ccccc34)nn2)c2ccc(Br)c(O)c21
**6b4**	10	0.640	aliphatic hydroxylation	O=C1C(=O)N(C(O)c2cn(CCCOc3cc(=O)oc4ccccc34)nn2)c2ccc(Br)cc21
**6b4**	10	0.640	*N*-dealkylation	O=Cc1cn(CCCOc2cc(=O)oc3ccccc23)nn1
**6b4**	10	0.640	*N*-dealkylation	O=C1Nc2ccc(Br)cc2C1=O
**6b4**	10	0.640	*N*-oxidation	O=C1C(=O)[N+]([O-])(Cc2cn(CCCOc3cc(=O)oc4ccccc34)nn2)c2ccc(Br)cc21
**6b4**	14	0.540	oxidation of 4-substituted anisole to quinone	O=CC=C(OCCCn1cc(CN2C(=O)C(=O)c3cc(Br)ccc32)nn1)C1=CC(=O)C=CC1=O
**6b4**	15	0.500	aromatic hydroxylation	O=C1C(=O)N(Cc2cn(CCCOc3cc(=O)oc4ccccc34)nn2)c2cc(O)c(Br)cc21
**6b4**	16	0.410	aliphatic hydroxylation	O=C1C(=O)N(Cc2cn(CC(O)COc3cc(=O)oc4ccccc34)nn2)c2ccc(Br)cc21
**6b4**	17	0.340	*N*-oxidation	O=C1C(=O)N(CC2=C[N+]([O-])(CCCOc3cc(=O)oc4ccccc34)N=N2)c2ccc(Br)cc21
**6b4**	17	0.340	*N*-dealkylation	O=C1C(=O)N(Cc2c[nH]nn2)c2ccc(Br)cc21
**6b4**	17	0.340	aliphatic hydroxylation	O=C1C(=O)N(Cc2cn(C(O)CCOc3cc(=O)oc4ccccc34)nn2)c2ccc(Br)cc21
**6b4**	17	0.340	*N*-dealkylation	O=CCCOc1cc(=O)oc2ccccc12
**6b4**	21	0.310	*N*-oxidation	O=C1C(=O)N(Cc2cn(CCCOc3cc(=O)oc4ccccc34)n[n+]2[O-])c2ccc(Br)cc21
**6b4**	22	0.184	alkyl dehydrogenation	O=C1C(=O)N(Cc2cn(CC=COc3cc(=O)oc4ccccc34)nn2)c2ccc(Br)cc21
**6b4**	23	0.160	epoxidation	O=C1C(=O)N(Cc2cn(CCCOC34OC3C(=O)Oc3ccccc34)nn2)c2ccc(Br)cc21
**6b4**	24	0.140	aromatic hydroxylation	O=C1C(=O)N(Cc2nnn(CCCOc3cc(=O)oc4ccccc34)c2O)c2ccc(Br)cc21
**6b4**	25	0.128	dehydration of N-C bond	O=C1C(=O)[N+](=Cc2cn(CCCOc3cc(=O)oc4ccccc34)nn2)c2ccc(Br)cc21
**6b4**	26	0.100	oxidative dehalogenation benzyl	O=C1C(=O)N(Cc2cn(CCCOc3cc(=O)oc4ccccc34)nn2)c2ccc(O)cc21
**6b4**	27	0.082	alkyl dehydrogenation	O=C1C(=O)N(Cc2cn(C=CCOc3cc(=O)oc4ccccc34)nn2)c2ccc(Br)cc21
**6b5 — Parent SMILES:** O=C1C(=O)N(Cc2cn(CCCOC3=CC(=O)Oc4ccccc34)nn2)c2ccc(I)cc21				
**6b5**	1	2.700	aromatic hydroxylation	O=C1C(=O)N(Cc2cn(CCCOc3cc(=O)oc4cc(O)ccc34)nn2)c2ccc(I)cc21

Compound	Rank	Priority score	Predicted transformation	Predicted metabolite SMILES
**6b5**	2	1.660	aromatic hydroxylation	O=C1C(=O)N(Cc2cn(CCCOc3cc(=O)oc4ccc(O)cc34)nn2)c2ccc(I)cc21
**6b5**	3	1.325	aromatic hydroxylation	O=C1C(=O)N(Cc2cn(CCCOc3cc(=O)oc4c(O)cccc34)nn2)c2ccc(I)cc21
**6b5**	4	0.920	aliphatic hydroxylation	O=C1C(=O)N(Cc2cn(CCC(O)Oc3cc(=O)oc4ccccc34)nn2)c2ccc(I)cc21
**6b5**	4	0.920	oxidative ether cleavage to one alcohol and one aldehyde/ketone	O=c1cc(O)c2ccccc2o1
**6b5**	4	0.920	oxidative ether cleavage to one alcohol and one aldehyde/ketone	O=CCCn1cc(CN2C(=O)C(=O)c3cc(I)ccc32)nn1
**6b5**	7	0.900	aromatic hydroxylation	O=C1C(=O)N(Cc2cn(CCCOc3cc(=O)oc4ccccc34)nn2)c2c(O)cc(I)cc21
**6b5**	8	0.660	aromatic hydroxylation	O=C1C(=O)N(Cc2cn(CCCOc3cc(=O)oc4cccc(O)c34)nn2)c2ccc(I)cc21
**6b5**	9	0.640	*N*-dealkylation	O=Cc1cn(CCCOc2cc(=O)oc3ccccc23)nn1
**6b5**	9	0.640	*N*-dealkylation	O=C1Nc2ccc(I)cc2C1=O
**6b5**	9	0.640	*N*-oxidation	O=C1C(=O)[N+]([O-])(Cc2cn(CCCOc3cc(=O)oc4ccccc34)nn2)c2ccc(I)cc21
**6b5**	9	0.640	aliphatic hydroxylation	O=C1C(=O)N(C(O)c2cn(CCCOc3cc(=O)oc4ccccc34)nn2)c2ccc(I)cc21
**6b5**	13	0.560	aromatic hydroxylation	O=C1C(=O)N(Cc2cn(CCCOc3cc(=O)oc4ccccc34)nn2)c2ccc(I)c(O)c21
**6b5**	14	0.540	oxidation of 4-substituted anisole to quinone	O=CC=C(OCCCn1cc(CN2C(=O)C(=O)c3cc(I)ccc32)nn1)C1=CC(=O)C=CC1=O
**6b5**	15	0.520	aromatic hydroxylation	O=C1C(=O)N(Cc2cn(CCCOc3cc(=O)oc4ccccc34)nn2)c2cc(O)c(I)cc21
**6b5**	16	0.400	aliphatic hydroxylation	O=C1C(=O)N(Cc2cn(CC(O)COc3cc(=O)oc4ccccc34)nn2)c2ccc(I)cc21
**6b5**	17	0.340	*N*-oxidation	O=C1C(=O)N(CC2=C[N+]([O-])(CCCOc3cc(=O)oc4ccccc34)N=N2)c2ccc(I)cc21
**6b5**	17	0.340	*N*-dealkylation	O=C1C(=O)N(Cc2c[nH]nn2)c2ccc(I)cc21
**6b5**	17	0.340	*N*-dealkylation	O=CCCOc1cc(=O)oc2ccccc12
**6b5**	17	0.340	aliphatic hydroxylation	O=C1C(=O)N(Cc2cn(C(O)CCOc3cc(=O)oc4ccccc34)nn2)c2ccc(I)cc21
**6b5**	21	0.300	*N*-oxidation	O=C1C(=O)N(Cc2cn(CCCOc3cc(=O)oc4ccccc34)n[n+]2[O-])c2ccc(I)cc21
**6b5**	22	0.238	oxidative dehalogenation benzyl	O=C1C(=O)N(Cc2cn(CCCOc3cc(=O)oc4ccccc34)nn2)c2ccc(O)cc21
**6b5**	23	0.184	alkyl dehydrogenation	O=C1C(=O)N(Cc2cn(CC=COc3cc(=O)oc4ccccc34)nn2)c2ccc(I)cc21
**6b5**	24	0.160	epoxidation	O=C1C(=O)N(Cc2cn(CCCOC34OC3C(=O)Oc3ccccc34)nn2)c2ccc(I)cc21
**6b5**	25	0.130	aromatic hydroxylation	O=C1C(=O)N(Cc2nnn(CCCOc3cc(=O)oc4ccccc34)c2O)c2ccc(I)cc21
**6b5**	26	0.128	dehydration of N-C bond	O=C1C(=O)[N+](=Cc2cn(CCCOc3cc(=O)oc4ccccc34)nn2)c2ccc(I)cc21

Compound	Rank	Priority score	Predicted transformation	Predicted metabolite SMILES
**6b5**	27	0.080	alkyl dehydrogenation	O=C1C(=O)N(Cc2cn(C=CCOc3cc(=O)oc4ccccc34)nn2)c2ccc(I)cc21
**6b6 — Parent SMILES:** O=C1C(=O)N(Cc2cn(CCCOC3=CC(=O)Oc4ccccc34)nn2)c2ccc([N+](=O)[O-])cc21				
**6b6**	1	2.640	aromatic hydroxylation	O=C1C(=O)N(Cc2cn(CCCOc3cc(=O)oc4cc(O)ccc34)nn2)c2ccc([N+](=O)[O-])cc21
**6b6**	2	1.650	aromatic hydroxylation	O=C1C(=O)N(Cc2cn(CCCOc3cc(=O)oc4ccc(O)cc34)nn2)c2ccc([N+](=O)[O-])cc21
**6b6**	3	1.325	aromatic hydroxylation	O=C1C(=O)N(Cc2cn(CCCOc3cc(=O)oc4c(O)cccc34)nn2)c2ccc([N+](=O)[O-])cc21
**6b6**	4	0.890	oxidative ether cleavage to one alcohol and one aldehyde/ketone	O=CCCn1cc(CN2C(=O)C(=O)c3cc([N+](=O)[O-])ccc32)nn1
**6b6**	4	0.890	oxidative ether cleavage to one alcohol and one aldehyde/ketone	O=c1cc(O)c2ccccc2o1
**6b6**	4	0.890	aliphatic hydroxylation	O=C1C(=O)N(Cc2cn(CCC(O)Oc3cc(=O)oc4ccccc34)nn2)c2ccc([N+](=O)[O-])cc21
**6b6**	7	0.850	aromatic hydroxylation	O=C1C(=O)N(Cc2cn(CCCOc3cc(=O)oc4ccccc34)nn2)c2ccc([N+](=O)[O-])c(O)c21
**6b6**	8	0.800	aromatic hydroxylation	O=C1C(=O)N(Cc2cn(CCCOc3cc(=O)oc4ccccc34)nn2)c2cc(O)c([N+](=O)[O-])cc21
**6b6**	9	0.680	aromatic hydroxylation	O=C1C(=O)N(Cc2cn(CCCOc3cc(=O)oc4cccc(O)c34)nn2)c2ccc([N+](=O)[O-])cc21
**6b6**	10	0.630	aliphatic hydroxylation	O=C1C(=O)N(C(O)c2cn(CCCOc3cc(=O)oc4ccccc34)nn2)c2ccc([N+](=O)[O-])cc21
**6b6**	10	0.630	*N*-dealkylation	O=Cc1cn(CCCOc2cc(=O)oc3ccccc23)nn1
**6b6**	10	0.630	*N*-dealkylation	O=C1Nc2ccc([N+](=O)[O-])cc2C1=O
**6b6**	10	0.630	*N*-oxidation	O=C1C(=O)[N+]([O-])(Cc2cn(CCCOc3cc(=O)oc4ccccc34)nn2)c2ccc([N+](=O)[O-])cc21
**6b6**	14	0.625	aromatic hydroxylation	O=C1C(=O)N(Cc2cn(CCCOc3cc(=O)oc4ccccc34)nn2)c2c(O)cc([N+](=O)[O-])cc21
**6b6**	16	0.528	oxidation of 4-substituted anisole to quinone	O=CC=C(OCCCn1cc(CN2C(=O)C(=O)c3cc([N+](=O)[O-])ccc32)nn1)C1=CC(=O)C=CC1=O
**6b6**	17	0.440	aliphatic hydroxylation	O=C1C(=O)N(Cc2cn(CC(O)COc3cc(=O)oc4ccccc34)nn2)c2ccc([N+](=O)[O-])cc21
**6b6**	18	0.320	*N*-dealkylation	O=C1C(=O)N(Cc2c[nH]nn2)c2ccc([N+](=O)[O-])cc21
**6b6**	18	0.320	aliphatic hydroxylation	O=C1C(=O)N(Cc2cn(C(O)CCOc3cc(=O)oc4ccccc34)nn2)c2ccc([N+](=O)[O-])cc21
**6b6**	18	0.320	*N*-dealkylation	O=CCCOc1cc(=O)oc2ccccc12
**6b6**	18	0.320	*N*-oxidation	O=C1C(=O)N(CC2=C[N+]([O-])(CCCOc3cc(=O)oc4ccccc34)N=N2)c2ccc([N+](=O)[O-])cc21
**6b6**	22	0.290	*N*-oxidation	O=C1C(=O)N(Cc2cn(CCCOc3cc(=O)oc4ccccc34)n[n+]2[O-])c2ccc([N+](=O)[O-])cc21
**6b6**	23	0.178	alkyl dehydrogenation	O=C1C(=O)N(Cc2cn(CC=COc3cc(=O)oc4ccccc34)nn2)c2ccc([N+](=O)[O-])cc21

Compound	Rank	Priority score	Predicted transformation	Predicted metabolite SMILES
**6b6**	24	0.160	epoxidation	O=C1C(=O)N(Cc2cn(CCCOC34OC3C(=O)Oc3ccccc34)nn2)c2ccc([N+](=O)[O-])cc21
**6b6**	25	0.130	aromatic hydroxylation	O=C1C(=O)N(Cc2nnn(CCCOc3cc(=O)oc4ccccc34)c2O)c2ccc([N+](=O)[O-])cc21
**6b6**	26	0.126	dehydration of N-C bond	O=C1C(=O)[N+](=Cc2cn(CCCOc3cc(=O)oc4ccccc34)nn2)c2ccc([N+](=O)[O-])cc21
**6b6**	27	0.112	reduction of nitro group	Nc1ccc2c(c1)C(=O)C(=O)N2Cc1cn(CCCOc2cc(=O)oc3ccccc23)nn1
**6b6**	28	0.088	alkyl dehydrogenation	O=C1C(=O)N(Cc2cn(C=CCOc3cc(=O)oc4ccccc34)nn2)c2ccc([N+](=O)[O-])cc21
**6b7 — Parent SMILES:** O=C1C(=O)N(Cc2cn(CCCOC3=CC(=O)Oc4ccccc34)nn2)c2ccc(C)cc21				
**6b7**	1	3.460	aliphatic hydroxylation	O=C1C(=O)N(Cc2cn(CCCOc3cc(=O)oc4ccccc34)nn2)c2ccc(CO)cc21
**6b7**	2	2.640	aromatic hydroxylation	Cc1ccc2c(c1)C(=O)C(=O)N2Cc1cn(CCCOc2cc(=O)oc3cc(O)ccc23)nn1
**6b7**	3	1.650	aromatic hydroxylation	Cc1ccc2c(c1)C(=O)C(=O)N2Cc1cn(CCCOc2cc(=O)oc3ccc(O)cc23)nn1
**6b7**	4	1.325	aromatic hydroxylation	Cc1ccc2c(c1)C(=O)C(=O)N2Cc1cn(CCCOc2cc(=O)oc3c(O)cccc23)nn1
**6b7**	5	0.890	oxidative ether cleavage to one alcohol and one aldehyde/ketone	Cc1ccc2c(c1)C(=O)C(=O)N2Cc1cn(CCC=O)nn1
**6b7**	5	0.890	oxidative ether cleavage to one alcohol and one aldehyde/ketone	O=c1cc(O)c2ccccc2o1
**6b7**	5	0.890	aliphatic hydroxylation	Cc1ccc2c(c1)C(=O)C(=O)N2Cc1cn(CCC(O)Oc2cc(=O)oc3ccccc23)nn1
**6b7**	8	0.710	aromatic hydroxylation	Cc1cc2c(cc1O)N(Cc1cn(CCCOc3cc(=O)oc4ccccc34)nn1)C(=O)C2=O
**6b7**	9	0.680	aromatic hydroxylation	Cc1ccc2c(c1)C(=O)C(=O)N2Cc1cn(CCCOc2cc(=O)oc3cccc(O)c23)nn1
**6b7**	10	0.610	aromatic hydroxylation	Cc1cc(O)c2c(c1)C(=O)C(=O)N2Cc1cn(CCCOc2cc(=O)oc3ccccc23)nn1
**6b7**	11	0.600	aliphatic hydroxylation	Cc1ccc2c(c1)C(=O)C(=O)N2C(O)c1cn(CCCOc2cc(=O)oc3ccccc23)nn1
**6b7**	11	0.600	*N*-dealkylation	O=Cc1cn(CCCOc2cc(=O)oc3ccccc23)nn1
**6b7**	11	0.600	*N*-oxidation	Cc1ccc2c(c1)C(=O)C(=O)[N+]2([O-])Cc1cn(CCCOc2cc(=O)oc3ccccc23)nn1
**6b7**	11	0.600	*N*-dealkylation	Cc1ccc2c(c1)C(=O)C(=O)N2
**6b7**	15	0.580	aromatic hydroxylation	Cc1ccc2c(c1O)C(=O)C(=O)N2Cc1cn(CCCOc2cc(=O)oc3ccccc23)nn1
**6b7**	16	0.528	oxidation of 4-substituted anisole to quinone	Cc1ccc2c(c1)C(=O)C(=O)N2Cc1cn(CCCOC(=CC=O)C2=CC(=O)C=CC2=O)nn1
**6b7**	17	0.440	aliphatic hydroxylation	Cc1ccc2c(c1)C(=O)C(=O)N2Cc1cn(CC(O)COc2cc(=O)oc3ccccc23)nn1
**6b7**	18	0.320	*N*-dealkylation	Cc1ccc2c(c1)C(=O)C(=O)N2Cc1c[nH]nn1
**6b7**	18	0.320	*N*-oxidation	Cc1ccc2c(c1)C(=O)C(=O)N2CC1=C[N+]([O-])(CCCOc2cc(=O)oc3ccccc23)N=N1

Compound	Rank	Priority score	Predicted transformation	Predicted metabolite SMILES
**6b7**	18	0.320	aliphatic hydroxylation	Cc1ccc2c(c1)C(=O)C(=O)N2Cc1cn(C(O)CCOc2cc(=O)oc3ccccc23)nn1
**6b7**	18	0.320	*N*-dealkylation	O=CCCOc1cc(=O)oc2ccccc12
**6b7**	22	0.290	*N*-oxidation	Cc1ccc2c(c1)C(=O)C(=O)N2Cc1cn(CCCOc2cc(=O)oc3ccccc23)n[n+]1[O-]
**6b7**	23	0.178	alkyl dehydrogenation	Cc1ccc2c(c1)C(=O)C(=O)N2Cc1cn(CC=COc2cc(=O)oc3ccccc23)nn1
**6b7**	24	0.160	epoxidation	Cc1ccc2c(c1)C(=O)C(=O)N2Cc1cn(CCCOC23OC2C(=O)Oc2ccccc23)nn1
**6b7**	25	0.130	aromatic hydroxylation	Cc1ccc2c(c1)C(=O)C(=O)N2Cc1nnn(CCCOc2cc(=O)oc3ccccc23)c1O
**6b7**	26	0.120	dehydration of N-C bond	Cc1ccc2c(c1)C(=O)C(=O)[N+]2=Cc1cn(CCCOc2cc(=O)oc3ccccc23)nn1
**6b7**	27	0.088	alkyl dehydrogenation	Cc1ccc2c(c1)C(=O)C(=O)N2Cc1cn(C=CCOc2cc(=O)oc3ccccc23)nn1
**Series 6c**				
**6c1 — Parent SMILES:** O=C1C(=O)N(Cc2cn(CCCCOC3=CC(=O)Oc4ccccc34)nn2)c2ccccc21				
**6c1**	1	2.640	aromatic hydroxylation	O=C1C(=O)N(Cc2cn(CCCCOc3cc(=O)oc4cc(O)ccc34)nn2)c2ccccc21
**6c1**	2	2.440	aromatic hydroxylation	O=C1C(=O)N(Cc2cn(CCCCOc3cc(=O)oc4ccccc34)nn2)c2ccc(O)cc21
**6c1**	3	1.680	aromatic hydroxylation	O=C1C(=O)N(Cc2cn(CCCCOc3cc(=O)oc4ccc(O)cc34)nn2)c2ccccc21
**6c1**	4	1.365	aromatic hydroxylation	O=C1C(=O)N(Cc2cn(CCCCOc3cc(=O)oc4c(O)cccc34)nn2)c2ccccc21
**6c1**	5	1.210	aliphatic hydroxylation	O=C1C(=O)N(Cc2cn(CCCC(O)Oc3cc(=O)oc4ccccc34)nn2)c2ccccc21
**6c1**	5	1.210	oxidative ether cleavage to one alcohol and one aldehyde/ketone	O=CCCCn1cc(CN2C(=O)C(=O)c3ccccc32)nn1
**6c1**	5	1.210	oxidative ether cleavage to one alcohol and one aldehyde/ketone	O=c1cc(O)c2ccccc2o1
**6c1**	8	1.110	aromatic hydroxylation	O=C1C(=O)N(Cc2cn(CCCCOc3cc(=O)oc4ccccc34)nn2)c2c(O)cccc21
**6c1**	9	0.850	aromatic hydroxylation	O=C1C(=O)N(Cc2cn(CCCCOc3cc(=O)oc4ccccc34)nn2)c2cc(O)ccc21
**6c1**	10	0.720	aromatic hydroxylation	O=C1C(=O)N(Cc2cn(CCCCOc3cc(=O)oc4cccc(O)c34)nn2)c2ccccc21
**6c1**	11	0.670	aromatic hydroxylation	O=C1C(=O)N(Cc2cn(CCCCOc3cc(=O)oc4ccccc34)nn2)c2cccc(O)c21
**6c1**	12	0.660	aliphatic hydroxylation	O=C1C(=O)N(C(O)c2cn(CCCCOc3cc(=O)oc4ccccc34)nn2)c2ccccc21
**6c1**	12	0.660	*N*-oxidation	O=C1C(=O)[N+]([O-])(Cc2cn(CCCCOc3cc(=O)oc4ccccc34)nn2)c2ccccc21
**6c1**	12	0.660	*N*-dealkylation	O=C1Nc2ccccc2C1=O
**6c1**	12	0.660	*N*-dealkylation	O=Cc1cn(CCCCOc2cc(=O)oc3ccccc23)nn1

Compound	Rank	Priority score	Predicted transformation	Predicted metabolite SMILES
**6c1**	16	0.528	oxidation of 4-substituted anisole to quinone	O=CC=C(OCCCCn1cc(CN2C(=O)C(=O)c3ccccc32)nn1)C1=CC(=O)C=CC1=O
**6c1**	17	0.390	aliphatic hydroxylation	O=C1C(=O)N(Cc2cn(CC(O)CCOc3cc(=O)oc4ccccc34)nn2)c2ccccc21
**6c1**	18	0.300	*N*-dealkylation	O=CCCCOc1cc(=O)oc2ccccc12
**6c1**	18	0.300	*N*-oxidation	O=C1C(=O)N(CC2=C[N+]([O-])(CCCCOc3cc(=O)oc4ccccc34)N=N2)c2ccccc21
**6c1**	18	0.300	*N*-dealkylation	O=C1C(=O)N(Cc2c[nH]nn2)c2ccccc21
**6c1**	21	0.270	aliphatic hydroxylation	O=C1C(=O)N(Cc2cn(CCC(O)COc3cc(=O)oc4ccccc34)nn2)c2ccccc21
**6c1**	22	0.260	*N*-oxidation	O=C1C(=O)N(Cc2cn(CCCCOc3cc(=O)oc4ccccc34)n[n+]2[O-])c2ccccc21
**6c1**	23	0.242	alkyl dehydrogenation	O=C1C(=O)N(Cc2cn(CCC=COc3cc(=O)oc4ccccc34)nn2)c2ccccc21
**6c1**	24	0.220	aliphatic hydroxylation	O=C1C(=O)N(Cc2cn(C(O)CCCOc3cc(=O)oc4ccccc34)nn2)c2ccccc21
**6c1**	25	0.200	epoxidation	O=C1C(=O)N(Cc2cn(CCCCOC34OC3C(=O)Oc3ccccc34)nn2)c2ccccc21
**6c1**	26	0.132	dehydration of N-C bond	O=C1C(=O)[N+](=Cc2cn(CCCCOc3cc(=O)oc4ccccc34)nn2)c2ccccc21
**6c1**	27	0.110	aromatic hydroxylation	O=C1C(=O)N(Cc2nnn(CCCCOc3cc(=O)oc4ccccc34)c2O)c2ccccc21
**6c1**	28	0.078	alkyl dehydrogenation	O=C1C(=O)N(Cc2cn(C=CCCOc3cc(=O)oc4ccccc34)nn2)c2ccccc21
**6c1**	28	0.078	alkyl dehydrogenation	O=C1C(=O)N(Cc2cn(CC=CCOc3cc(=O)oc4ccccc34)nn2)c2ccccc21
**6c2 — Parent SMILES:** O=C1C(=O)N(Cc2cn(CCCCOC3=CC(=O)Oc4ccccc34)nn2)c2ccc(F)cc21				
**6c2**	1	2.640	aromatic hydroxylation	O=C1C(=O)N(Cc2cn(CCCCOc3cc(=O)oc4cc(O)ccc34)nn2)c2ccc(F)cc21
**6c2**	2	1.680	aromatic hydroxylation	O=C1C(=O)N(Cc2cn(CCCCOc3cc(=O)oc4ccc(O)cc34)nn2)c2ccc(F)cc21
**6c2**	3	1.365	aromatic hydroxylation	O=C1C(=O)N(Cc2cn(CCCCOc3cc(=O)oc4c(O)cccc34)nn2)c2ccc(F)cc21
**6c2**	4	1.210	aliphatic hydroxylation	O=C1C(=O)N(Cc2cn(CCCC(O)Oc3cc(=O)oc4ccccc34)nn2)c2ccc(F)cc21
**6c2**	4	1.210	oxidative ether cleavage to one alcohol and one aldehyde/ketone	O=c1cc(O)c2ccccc2o1
**6c2**	4	1.210	oxidative ether cleavage to one alcohol and one aldehyde/ketone	O=CCCCn1cc(CN2C(=O)C(=O)c3cc(F)ccc32)nn1
**6c2**	7	1.090	aromatic hydroxylation	O=C1C(=O)N(Cc2cn(CCCCOc3cc(=O)oc4ccccc34)nn2)c2c(O)cc(F)cc21
**6c2**	8	0.940	aromatic hydroxylation	O=C1C(=O)N(Cc2cn(CCCCOc3cc(=O)oc4ccccc34)nn2)c2cc(O)c(F)cc21
**6c2**	9	0.720	aromatic hydroxylation	O=C1C(=O)N(Cc2cn(CCCCOc3cc(=O)oc4cccc(O)c34)nn2)c2ccc(F)cc21
**6c2**	10	0.670	aromatic hydroxylation	O=C1C(=O)N(Cc2cn(CCCCOc3cc(=O)oc4ccccc34)nn2)c2ccc(F)c(O)c21

Compo und	Rank	Priority score	Predicted transformation	Predicted metabolite SMILES
**6c2**	11	0.660	aliphatic hydroxylation	O=C1C(=O)N(C(O)c2cn(CCCCOc3cc(=O)oc4ccccc34)nn2)c2ccc(F)cc21
**6c2**	11	0.660	*N*-oxidation	O=C1C(=O)[N+]([O-])(Cc2cn(CCCCOc3cc(=O)oc4ccccc34)nn2)c2ccc(F)cc21
**6c2**	11	0.660	*N*-dealkylation	O=C1Nc2ccc(F)cc2C1=O
**6c2**	11	0.660	*N*-dealkylation	O=Cc1cn(CCCCOc2cc(=O)oc3ccccc23)nn1
**6c2**	15	0.528	oxidation of 4-substituted anisole to quinone	O=CC=C(OCCCCn1cc(CN2C(=O)C(=O)c3cc(F)ccc32)nn1)C1=CC(=O)C=CC1=O
**6c2**	16	0.390	aliphatic hydroxylation	O=C1C(=O)N(Cc2cn(CC(O)CCOc3cc(=O)oc4ccccc34)nn2)c2ccc(F)cc21
**6c2**	17	0.300	*N*-oxidation	O=C1C(=O)N(CC2=C[N+]([O-])(CCCCOc3cc(=O)oc4ccccc34)N=N2)c2ccc(F)cc21
**6c2**	17	0.300	*N*-dealkylation	O=CCCCOc1cc(=O)oc2ccccc12
**6c2**	17	0.300	*N*-dealkylation	O=C1C(=O)N(Cc2c[nH]nn2)c2ccc(F)cc21
**6c2**	20	0.270	aliphatic hydroxylation	O=C1C(=O)N(Cc2cn(CCC(O)COc3cc(=O)oc4ccccc34)nn2)c2ccc(F)cc21
**6c2**	21	0.260	*N*-oxidation	O=C1C(=O)N(Cc2cn(CCCCOc3cc(=O)oc4ccccc34)n[n+]2[O-])c2ccc(F)cc21
**6c2**	22	0.242	alkyl dehydrogenation	O=C1C(=O)N(Cc2cn(CCC=COc3cc(=O)oc4ccccc34)nn2)c2ccc(F)cc21
**6c2**	23	0.220	aliphatic hydroxylation	O=C1C(=O)N(Cc2cn(C(O)CCCOc3cc(=O)oc4ccccc34)nn2)c2ccc(F)cc21
**6c2**	24	0.200	epoxidation	O=C1C(=O)N(Cc2cn(CCCCOC34OC3C(=O)Oc3ccccc34)nn2)c2ccc(F)cc21
**6c2**	25	0.132	dehydration of N-C bond	O=C1C(=O)[N+](=Cc2cn(CCCCOc3cc(=O)oc4ccccc34)nn2)c2ccc(F)cc21
**6c2**	26	0.110	aromatic hydroxylation	O=C1C(=O)N(Cc2nnn(CCCCOc3cc(=O)oc4ccccc34)c2O)c2ccc(F)cc21
**6c2**	27	0.092	oxidative dehalogenation benzyl	O=C1C(=O)N(Cc2cn(CCCCOc3cc(=O)oc4ccccc34)nn2)c2ccc(O)cc21
**6c2**	28	0.078	alkyl dehydrogenation	O=C1C(=O)N(Cc2cn(C=CCCOc3cc(=O)oc4ccccc34)nn2)c2ccc(F)cc21
**6c2**	28	0.078	alkyl dehydrogenation	O=C1C(=O)N(Cc2cn(CC=CCOc3cc(=O)oc4ccccc34)nn2)c2ccc(F)cc21
**6c3 — Parent SMILES:** O=C1C(=O)N(Cc2cn(CCCCOC3=CC(=O)Oc4ccccc34)nn2)c2ccc(Cl)cc21				
**6c3**	1	2.640	aromatic hydroxylation	O=C1C(=O)N(Cc2cn(CCCCOc3cc(=O)oc4cc(O)ccc34)nn2)c2ccc(Cl)cc21
**6c3**	2	1.680	aromatic hydroxylation	O=C1C(=O)N(Cc2cn(CCCCOc3cc(=O)oc4ccc(O)cc34)nn2)c2ccc(Cl)cc21
**6c3**	3	1.365	aromatic hydroxylation	O=C1C(=O)N(Cc2cn(CCCCOc3cc(=O)oc4c(O)cccc34)nn2)c2ccc(Cl)cc21
**6c3**	4	1.210	aliphatic hydroxylation	O=C1C(=O)N(Cc2cn(CCCC(O)Oc3cc(=O)oc4ccccc34)nn2)c2ccc(Cl)cc21
**6c3**	4	1.210	oxidative ether cleavage to one alcohol and one aldehyde/ketone	O=CCCCn1cc(CN2C(=O)C(=O)c3cc(Cl)ccc32)nn1

Compound	Rank	Priority score	Predicted transformation	Predicted metabolite SMILES
**6c3**	4	1.210	oxidative ether cleavage to one alcohol and one aldehyde/ketone	O=c1cc(O)c2ccccc2o1
**6c3**	7	0.940	aromatic hydroxylation	O=C1C(=O)N(Cc2cn(CCCCOc3cc(=O)oc4ccccc34)nn2)c2c(O)cc(Cl)cc21
**6c3**	8	0.720	aromatic hydroxylation	O=C1C(=O)N(Cc2cn(CCCCOc3cc(=O)oc4cccc(O)c34)nn2)c2ccc(Cl)cc21
**6c3**	9	0.660	*N*-oxidation	O=C1C(=O)[N+]([O-])(Cc2cn(CCCCOc3cc(=O)oc4ccccc34)nn2)c2ccc(Cl)cc21
**6c3**	9	0.660	aliphatic hydroxylation	O=C1C(=O)N(C(O)c2cn(CCCCOc3cc(=O)oc4ccccc34)nn2)c2ccc(Cl)cc21
**6c3**	9	0.660	*N*-dealkylation	O=C1Nc2ccc(Cl)cc2C1=O
**6c3**	9	0.660	*N*-dealkylation	O=Cc1cn(CCCCOc2cc(=O)oc3ccccc23)nn1
**6c3**	13	0.640	aromatic hydroxylation	O=C1C(=O)N(Cc2cn(CCCCOc3cc(=O)oc4ccccc34)nn2)c2ccc(Cl)c(O)c21
**6c3**	14	0.560	aromatic hydroxylation	O=C1C(=O)N(Cc2cn(CCCCOc3cc(=O)oc4ccccc34)nn2)c2cc(O)c(Cl)cc21
**6c3**	15	0.528	oxidation of 4-substituted anisole to quinone	O=CC=C(OCCCCn1cc(CN2C(=O)C(=O)c3cc(Cl)ccc32)nn1)C1=CC(=O)C=CC1=O
**6c3**	16	0.390	aliphatic hydroxylation	O=C1C(=O)N(Cc2cn(CC(O)CCOc3cc(=O)oc4ccccc34)nn2)c2ccc(Cl)cc21
**6c3**	17	0.300	*N*-oxidation	O=C1C(=O)N(CC2=C[N+]([O-])(CCCCOc3cc(=O)oc4ccccc34)N=N2)c2ccc(Cl)cc21
**6c3**	17	0.300	*N*-dealkylation	O=CCCCOc1cc(=O)oc2ccccc12
**6c3**	17	0.300	*N*-dealkylation	O=C1C(=O)N(Cc2c[nH]nn2)c2ccc(Cl)cc21
**6c3**	20	0.270	aliphatic hydroxylation	O=C1C(=O)N(Cc2cn(CCC(O)COc3cc(=O)oc4ccccc34)nn2)c2ccc(Cl)cc21
**6c3**	21	0.260	*N*-oxidation	O=C1C(=O)N(Cc2cn(CCCCOc3cc(=O)oc4ccccc34)n[n+]2[O-])c2ccc(Cl)cc21
**6c3**	22	0.242	alkyl dehydrogenation	O=C1C(=O)N(Cc2cn(CCC=COc3cc(=O)oc4ccccc34)nn2)c2ccc(Cl)cc21
**6c3**	23	0.220	aliphatic hydroxylation	O=C1C(=O)N(Cc2cn(C(O)CCCOc3cc(=O)oc4ccccc34)nn2)c2ccc(Cl)cc21
**6c3**	24	0.200	epoxidation	O=C1C(=O)N(Cc2cn(CCCCOC34OC3C(=O)Oc3ccccc34)nn2)c2ccc(Cl)cc21
**6c3**	25	0.132	dehydration of N-C bond	O=C1C(=O)[N+](=Cc2cn(CCCCOc3cc(=O)oc4ccccc34)nn2)c2ccc(Cl)cc21
**6c3**	26	0.110	aromatic hydroxylation	O=C1C(=O)N(Cc2nnn(CCCCOc3cc(=O)oc4ccccc34)c2O)c2ccc(Cl)cc21
**6c3**	27	0.078	alkyl dehydrogenation	O=C1C(=O)N(Cc2cn(C=CCCOc3cc(=O)oc4ccccc34)nn2)c2ccc(Cl)cc21
**6c3**	27	0.078	alkyl dehydrogenation	O=C1C(=O)N(Cc2cn(CC=CCOc3cc(=O)oc4ccccc34)nn2)c2ccc(Cl)cc21
**6c3**	29	0.038	oxidative dehalogenation benzyl	O=C1C(=O)N(Cc2cn(CCCCOc3cc(=O)oc4ccccc34)nn2)c2ccc(O)cc21
**6c4 — Parent SMILES:** O=C1C(=O)N(Cc2cn(CCCCOC3=CC(=O)Oc4ccccc34)nn2)c2ccc(Br)cc21				

Compound	Rank	Priority score	Predicted transformation	Predicted metabolite SMILES
**6c4**	1	2.640	aromatic hydroxylation	O=C1C(=O)N(Cc2cn(CCCCOc3cc(=O)oc4cc(O)ccc34)nn2)c2ccc(Br)cc21
**6c4**	2	1.680	aromatic hydroxylation	O=C1C(=O)N(Cc2cn(CCCCOc3cc(=O)oc4ccc(O)cc34)nn2)c2ccc(Br)cc21
**6c4**	3	1.365	aromatic hydroxylation	O=C1C(=O)N(Cc2cn(CCCCOc3cc(=O)oc4c(O)cccc34)nn2)c2ccc(Br)cc21
**6c4**	4	1.210	oxidative ether cleavage to one alcohol and one aldehyde/ketone	O=CCCCn1cc(CN2C(=O)C(=O)c3cc(Br)ccc32)nn1
**6c4**	4	1.210	aliphatic hydroxylation	O=C1C(=O)N(Cc2cn(CCCC(O)Oc3cc(=O)oc4ccccc34)nn2)c2ccc(Br)cc21
**6c4**	4	1.210	oxidative ether cleavage to one alcohol and one aldehyde/ketone	O=c1cc(O)c2ccccc2o1
**6c4**	7	0.940	aromatic hydroxylation	O=C1C(=O)N(Cc2cn(CCCCOc3cc(=O)oc4ccccc34)nn2)c2c(O)cc(Br)cc21
**6c4**	8	0.720	aromatic hydroxylation	O=C1C(=O)N(Cc2cn(CCCCOc3cc(=O)oc4cccc(O)c34)nn2)c2ccc(Br)cc21
**6c4**	9	0.650	*N*-dealkylation	O=Cc1cn(CCCCOc2cc(=O)oc3ccccc23)nn1
**6c4**	9	0.650	aliphatic hydroxylation	O=C1C(=O)N(C(O)c2cn(CCCCOc3cc(=O)oc4ccccc34)nn2)c2ccc(Br)cc21
**6c4**	9	0.650	*N*-dealkylation	O=C1Nc2ccc(Br)cc2C1=O
**6c4**	9	0.650	*N*-oxidation	O=C1C(=O)[N+]([O-])(Cc2cn(CCCCOc3cc(=O)oc4ccccc34)nn2)c2ccc(Br)cc21
**6c4**	13	0.610	aromatic hydroxylation	O=C1C(=O)N(Cc2cn(CCCCOc3cc(=O)oc4ccccc34)nn2)c2ccc(Br)c(O)c21
**6c4**	14	0.528	oxidation of 4-substituted anisole to quinone	O=CC=C(OCCCCn1cc(CN2C(=O)C(=O)c3cc(Br)ccc32)nn1)C1=CC(=O)C=CC1=O
**6c4**	15	0.480	aromatic hydroxylation	O=C1C(=O)N(Cc2cn(CCCCOc3cc(=O)oc4ccccc34)nn2)c2cc(O)c(Br)cc21
**6c4**	16	0.390	aliphatic hydroxylation	O=C1C(=O)N(Cc2cn(CC(O)CCOc3cc(=O)oc4ccccc34)nn2)c2ccc(Br)cc21
**6c4**	17	0.300	*N*-oxidation	O=C1C(=O)N(CC2=C[N+]([O-])(CCCCOc3cc(=O)oc4ccccc34)N=N2)c2ccc(Br)cc21
**6c4**	17	0.300	*N*-dealkylation	O=CCCCOc1cc(=O)oc2ccccc12
**6c4**	17	0.300	*N*-dealkylation	O=C1C(=O)N(Cc2c[nH]nn2)c2ccc(Br)cc21
**6c4**	20	0.270	aliphatic hydroxylation	O=C1C(=O)N(Cc2cn(CCC(O)COc3cc(=O)oc4ccccc34)nn2)c2ccc(Br)cc21
**6c4**	21	0.260	*N*-oxidation	O=C1C(=O)N(Cc2cn(CCCCOc3cc(=O)oc4ccccc34)n[n+]2[O-])c2ccc(Br)cc21
**6c4**	22	0.242	alkyl dehydrogenation	O=C1C(=O)N(Cc2cn(CCC=COc3cc(=O)oc4ccccc34)nn2)c2ccc(Br)cc21
**6c4**	23	0.220	aliphatic hydroxylation	O=C1C(=O)N(Cc2cn(C(O)CCCOc3cc(=O)oc4ccccc34)nn2)c2ccc(Br)cc21
**6c4**	24	0.200	epoxidation	O=C1C(=O)N(Cc2cn(CCCCOC34OC3C(=O)Oc3ccccc34)nn2)c2ccc(Br)cc21
**6c4**	25	0.130	dehydration of N-C bond	O=C1C(=O)[N+](=Cc2cn(CCCCOc3cc(=O)oc4ccccc34)nn2)c2ccc(Br)cc21

Compound	Rank	Priority score	Predicted transformation	Predicted metabolite SMILES
**6c4**	26	0.100	aromatic hydroxylation	O=C1C(=O)N(Cc2nnn(CCCCOc3cc(=O)oc4ccccc34)c2O)c2ccc(Br)cc21
**6c4**	27	0.098	oxidative dehalogenation benzyl	O=C1C(=O)N(Cc2cn(CCCCOc3cc(=O)oc4ccccc34)nn2)c2ccc(O)cc21
**6c4**	28	0.078	alkyl dehydrogenation	O=C1C(=O)N(Cc2cn(CC=CCOc3cc(=O)oc4ccccc34)nn2)c2ccc(Br)cc21
**6c4**	28	0.078	alkyl dehydrogenation	O=C1C(=O)N(Cc2cn(C=CCCOc3cc(=O)oc4ccccc34)nn2)c2ccc(Br)cc21
**6c5 — Parent SMILES:** O=C1C(=O)N(Cc2cn(CCCCOC3=CC(=O)Oc4ccccc34)nn2)c2ccc(I)cc21				
**6c5**	1	2.640	aromatic hydroxylation	O=C1C(=O)N(Cc2cn(CCCCOc3cc(=O)oc4cc(O)ccc34)nn2)c2ccc(I)cc21
**6c5**	2	1.680	aromatic hydroxylation	O=C1C(=O)N(Cc2cn(CCCCOc3cc(=O)oc4ccc(O)cc34)nn2)c2ccc(I)cc21
**6c5**	3	1.365	aromatic hydroxylation	O=C1C(=O)N(Cc2cn(CCCCOc3cc(=O)oc4c(O)cccc34)nn2)c2ccc(I)cc21
**6c5**	4	1.210	oxidative ether cleavage to one alcohol and one aldehyde/ketone	O=CCCCn1cc(CN2C(=O)C(=O)c3cc(I)ccc32)nn1
**6c5**	4	1.210	aliphatic hydroxylation	O=C1C(=O)N(Cc2cn(CCCC(O)Oc3cc(=O)oc4ccccc34)nn2)c2ccc(I)cc21
**6c5**	4	1.210	oxidative ether cleavage to one alcohol and one aldehyde/ketone	O=c1cc(O)c2ccccc2o1
**6c5**	7	0.890	aromatic hydroxylation	O=C1C(=O)N(Cc2cn(CCCCOc3cc(=O)oc4ccccc34)nn2)c2c(O)cc(I)cc21
**6c5**	8	0.720	aromatic hydroxylation	O=C1C(=O)N(Cc2cn(CCCCOc3cc(=O)oc4cccc(O)c34)nn2)c2ccc(I)cc21
**6c5**	9	0.640	*N*-dealkylation	O=C1Nc2ccc(I)cc2C1=O
**6c5**	9	0.640	*N*-dealkylation	O=Cc1cn(CCCCOc2cc(=O)oc3ccccc23)nn1
**6c5**	9	0.640	aliphatic hydroxylation	O=C1C(=O)N(C(O)c2cn(CCCCOc3cc(=O)oc4ccccc34)nn2)c2ccc(I)cc21
**6c5**	9	0.640	*N*-oxidation	O=C1C(=O)[N+]([O-])(Cc2cn(CCCCOc3cc(=O)oc4ccccc34)nn2)c2ccc(I)cc21
**6c5**	13	0.540	aromatic hydroxylation	O=C1C(=O)N(Cc2cn(CCCCOc3cc(=O)oc4ccccc34)nn2)c2ccc(I)c(O)c21
**6c5**	14	0.528	oxidation of 4-substituted anisole to quinone	O=CC=C(OCCCCn1cc(CN2C(=O)C(=O)c3cc(I)ccc32)nn1)C1=CC(=O)C=CC1=O
**6c5**	15	0.500	aromatic hydroxylation	O=C1C(=O)N(Cc2cn(CCCCOc3cc(=O)oc4ccccc34)nn2)c2cc(O)c(I)cc21
**6c5**	16	0.390	aliphatic hydroxylation	O=C1C(=O)N(Cc2cn(CC(O)CCOc3cc(=O)oc4ccccc34)nn2)c2ccc(I)cc21
**6c5**	17	0.290	*N*-dealkylation	O=C1C(=O)N(Cc2c[nH]nn2)c2ccc(I)cc21
**6c5**	17	0.290	*N*-oxidation	O=C1C(=O)N(CC2=C[N+]([O-])(CCCCOc3cc(=O)oc4ccccc34)N=N2)c2ccc(I)cc21
**6c5**	17	0.290	*N*-dealkylation	O=CCCCOc1cc(=O)oc2ccccc12
**6c5**	20	0.270	aliphatic hydroxylation	O=C1C(=O)N(Cc2cn(CCC(O)COc3cc(=O)oc4ccccc34)nn2)c2ccc(I)cc21

Compound	Rank	Priority score	Predicted transformation	Predicted metabolite SMILES
**6c5**	21	0.250	*N*-oxidation	O=C1C(=O)N(Cc2cn(CCCCOc3cc(=O)oc4ccccc34)n[n+]2[O-])c2ccc(I)cc21
**6c5**	22	0.242	alkyl dehydrogenation	O=C1C(=O)N(Cc2cn(CCC=COc3cc(=O)oc4ccccc34)nn2)c2ccc(I)cc21
**6c5**	23	0.234	oxidative dehalogenation benzyl	O=C1C(=O)N(Cc2cn(CCCCOc3cc(=O)oc4ccccc34)nn2)c2ccc(O)cc21
**6c5**	24	0.220	aliphatic hydroxylation	O=C1C(=O)N(Cc2cn(C(O)CCCOc3cc(=O)oc4ccccc34)nn2)c2ccc(I)cc21
**6c5**	25	0.200	epoxidation	O=C1C(=O)N(Cc2cn(CCCCOC34OC3C(=O)Oc3ccccc34)nn2)c2ccc(I)cc21
**6c5**	26	0.128	dehydration of N-C bond	O=C1C(=O)[N+](=Cc2cn(CCCCOc3cc(=O)oc4ccccc34)nn2)c2ccc(I)cc21
**6c5**	27	0.090	aromatic hydroxylation	O=C1C(=O)N(Cc2nnn(CCCCOc3cc(=O)oc4ccccc34)c2O)c2ccc(I)cc21
**6c5**	28	0.078	alkyl dehydrogenation	O=C1C(=O)N(Cc2cn(C=CCCOc3cc(=O)oc4ccccc34)nn2)c2ccc(I)cc21
**6c5**	28	0.078	alkyl dehydrogenation	O=C1C(=O)N(Cc2cn(CC=CCOc3cc(=O)oc4ccccc34)nn2)c2ccc(I)cc21
**6c6 — Parent SMILES:** O=C1C(=O)N(Cc2cn(CCCCOC3=CC(=O)Oc4ccccc34)nn2)c2ccc([N+](=O)[O-])cc21				
**6c6**	1	2.600	aromatic hydroxylation	O=C1C(=O)N(Cc2cn(CCCCOc3cc(=O)oc4cc(O)ccc34)nn2)c2ccc([N+](=O)[O-])cc21
**6c6**	2	1.620	aromatic hydroxylation	O=C1C(=O)N(Cc2cn(CCCCOc3cc(=O)oc4ccc(O)cc34)nn2)c2ccc([N+](=O)[O-])cc21
**6c6**	3	1.335	aromatic hydroxylation	O=C1C(=O)N(Cc2cn(CCCCOc3cc(=O)oc4c(O)cccc34)nn2)c2ccc([N+](=O)[O-])cc21
**6c6**	4	1.200	oxidative ether cleavage to one alcohol and one aldehyde/ketone	O=CCCCn1cc(CN2C(=O)C(=O)c3cc([N+](=O)[O-])ccc32)nn1
**6c6**	4	1.200	oxidative ether cleavage to one alcohol and one aldehyde/ketone	O=c1cc(O)c2ccccc2o1
**6c6**	4	1.200	aliphatic hydroxylation	O=C1C(=O)N(Cc2cn(CCCC(O)Oc3cc(=O)oc4ccccc34)nn2)c2ccc([N+](=O)[O-])cc21
**6c6**	7	0.960	aromatic hydroxylation	O=C1C(=O)N(Cc2cn(CCCCOc3cc(=O)oc4cccc(O)c34)nn2)c2ccc([N+](=O)[O-])cc21
**6c6**	8	0.900	aromatic hydroxylation	O=C1C(=O)N(Cc2cn(CCCCOc3cc(=O)oc4ccccc34)nn2)c2ccc([N+](=O)[O-])c(O)c21
**6c6**	9	0.840	aromatic hydroxylation	O=C1C(=O)N(Cc2cn(CCCCOc3cc(=O)oc4ccccc34)nn2)c2cc(O)c([N+](=O)[O-])cc21
**6c6**	10	0.705	aromatic hydroxylation	O=C1C(=O)N(Cc2cn(CCCCOc3cc(=O)oc4ccccc34)nn2)c2c(O)cc([N+](=O)[O-])cc21
**6c6**	11	0.680	*N*-oxidation	O=C1C(=O)[N+]([O-])(Cc2cn(CCCCOc3cc(=O)oc4ccccc34)nn2)c2ccc([N+](=O)[O-])cc21
**6c6**	11	0.680	*N*-dealkylation	O=Cc1cn(CCCCOc2cc(=O)oc3ccccc23)nn1
**6c6**	11	0.680	aliphatic hydroxylation	O=C1C(=O)N(C(O)c2cn(CCCCOc3cc(=O)oc4ccccc34)nn2)c2ccc([N+](=O)[O-])cc21
**6c6**	11	0.680	*N*-dealkylation	O=C1Nc2ccc([N+](=O)[O-])cc2C1=O
**6c6**	16	0.520	oxidation of 4-substituted anisole to quinone	O=CC=C(OCCCCn1cc(CN2C(=O)C(=O)c3cc([N+](=O)[O-])ccc32)nn1)C1=CC(=O)C=CC1=O

Compound	Rank	Priority score	Predicted transformation	Predicted metabolite SMILES
**6c6**	17	0.420	aliphatic hydroxylation	O=C1C(=O)N(Cc2cn(CC(O)CCOc3cc(=O)oc4ccccc34)nn2)c2ccc([N+](=O)[O-])cc21
**6c6**	18	0.300	*N*-dealkylation	O=C1C(=O)N(Cc2c[nH]nn2)c2ccc([N+](=O)[O-])cc21
**6c6**	18	0.300	*N*-dealkylation	O=CCCCOc1cc(=O)oc2ccccc12
**6c6**	18	0.300	*N*-oxidation	O=C1C(=O)N(CC2=C[N+]([O-])(CCCCOc3cc(=O)oc4ccccc34)N=N2)c2ccc([N+](=O)[O-])cc21
**6c6**	21	0.280	aliphatic hydroxylation	O=C1C(=O)N(Cc2cn(CCC(O)COc3cc(=O)oc4ccccc34)nn2)c2ccc([N+](=O)[O-])cc21
**6c6**	22	0.260	*N*-oxidation	O=C1C(=O)N(Cc2cn(CCCCOc3cc(=O)oc4ccccc34)n[n+]2[O-])c2ccc([N+](=O)[O-])cc21
**6c6**	23	0.240	alkyl dehydrogenation	O=C1C(=O)N(Cc2cn(CCC=COc3cc(=O)oc4ccccc34)nn2)c2ccc([N+](=O)[O-])cc21
**6c6**	24	0.220	epoxidation	O=C1C(=O)N(Cc2cn(CCCCOC34OC3C(=O)Oc3ccccc34)nn2)c2ccc([N+](=O)[O-])cc21
**6c6**	25	0.210	aliphatic hydroxylation	O=C1C(=O)N(Cc2cn(C(O)CCCOc3cc(=O)oc4ccccc34)nn2)c2ccc([N+](=O)[O-])cc21
**6c6**	26	0.136	dehydration of N-C bond	O=C1C(=O)[N+](=Cc2cn(CCCCOc3cc(=O)oc4ccccc34)nn2)c2ccc([N+](=O)[O-])cc21
**6c6**	27	0.120	aromatic hydroxylation	O=C1C(=O)N(Cc2nnn(CCCCOc3cc(=O)oc4ccccc34)c2O)c2ccc([N+](=O)[O-])cc21
**6c6**	28	0.110	reduction of nitro group	Nc1ccc2c(c1)C(=O)C(=O)N2Cc1cn(CCCCOc2cc(=O)oc3ccccc23)nn1
**6c6**	29	0.084	alkyl dehydrogenation	O=C1C(=O)N(Cc2cn(CC=CCOc3cc(=O)oc4ccccc34)nn2)c2ccc([N+](=O)[O-])cc21
**6c6**	29	0.084	alkyl dehydrogenation	O=C1C(=O)N(Cc2cn(C=CCCOc3cc(=O)oc4ccccc34)nn2)c2ccc([N+](=O)[O-])cc21
**6c7 — Parent SMILES:** O=C1C(=O)N(Cc2cn(CCCCOC3=CC(=O)Oc4ccccc34)nn2)c2ccc(C)cc21				
**6c7**	1	3.410	aliphatic hydroxylation	O=C1C(=O)N(Cc2cn(CCCCOc3cc(=O)oc4ccccc34)nn2)c2ccc(CO)cc21
**6c7**	2	2.600	aromatic hydroxylation	Cc1ccc2c(c1)C(=O)C(=O)N2Cc1cn(CCCCOc2cc(=O)oc3cc(O)ccc23)nn1
**6c7**	3	1.620	aromatic hydroxylation	Cc1ccc2c(c1)C(=O)C(=O)N2Cc1cn(CCCCOc2cc(=O)oc3ccc(O)cc23)nn1
**6c7**	4	1.335	aromatic hydroxylation	Cc1ccc2c(c1)C(=O)C(=O)N2Cc1cn(CCCCOc2cc(=O)oc3c(O)cccc23)nn1
**6c7**	5	1.200	oxidative ether cleavage to one alcohol and one aldehyde/ketone	Cc1ccc2c(c1)C(=O)C(=O)N2Cc1cn(CCCC=O)nn1
**6c7**	5	1.200	aliphatic hydroxylation	Cc1ccc2c(c1)C(=O)C(=O)N2Cc1cn(CCCC(O)Oc2cc(=O)oc3ccccc23)nn1
**6c7**	5	1.200	oxidative ether cleavage to one alcohol and one aldehyde/ketone	O=c1cc(O)c2ccccc2o1
**6c7**	8	0.960	aromatic hydroxylation	Cc1ccc2c(c1)C(=O)C(=O)N2Cc1cn(CCCCOc2cc(=O)oc3cccc(O)c23)nn1
**6c7**	9	0.810	aromatic hydroxylation	Cc1cc2c(cc1O)N(Cc1cn(CCCCOc3cc(=O)oc4ccccc34)nn1)C(=O)C2=O
**6c7**	10	0.700	aromatic hydroxylation	Cc1cc(O)c2c(c1)C(=O)C(=O)N2Cc1cn(CCCCOc2cc(=O)oc3ccccc23)nn1

Compound	Rank	Priority score	Predicted transformation	Predicted metabolite SMILES
**6c7**	11	0.660	aromatic hydroxylation	Cc1ccc2c(c1O)C(=O)C(=O)N2Cc1cn(CCCCOc2cc(=O)oc3ccccc23)nn1
**6c7**	12	0.640	*N*-oxidation	Cc1ccc2c(c1)C(=O)C(=O)[N+]2([O-])Cc1cn(CCCCOc2cc(=O)oc3ccccc23)nn1
**6c7**	12	0.640	aliphatic hydroxylation	Cc1ccc2c(c1)C(=O)C(=O)N2C(O)c1cn(CCCCOc2cc(=O)oc3ccccc23)nn1
**6c7**	12	0.640	*N*-dealkylation	O=Cc1cn(CCCCOc2cc(=O)oc3ccccc23)nn1
**6c7**	12	0.640	*N*-dealkylation	Cc1ccc2c(c1)C(=O)C(=O)N2
**6c7**	16	0.520	oxidation of 4-substituted anisole to quinone	Cc1ccc2c(c1)C(=O)C(=O)N2Cc1cn(CCCCOC(=CC=O)C2=CC(=O)C=CC2=O)nn1
**6c7**	17	0.420	aliphatic hydroxylation	Cc1ccc2c(c1)C(=O)C(=O)N2Cc1cn(CC(O)CCOc2cc(=O)oc3ccccc23)nn1
**6c7**	18	0.300	*N*-dealkylation	O=CCCCOc1cc(=O)oc2ccccc12
**6c7**	18	0.300	*N*-dealkylation	Cc1ccc2c(c1)C(=O)C(=O)N2Cc1c[nH]nn1
**6c7**	18	0.300	*N*-oxidation	Cc1ccc2c(c1)C(=O)C(=O)N2CC1=C[N+]([O-])(CCCCOc2cc(=O)oc3ccccc23)N=N1
**6c7**	21	0.280	aliphatic hydroxylation	Cc1ccc2c(c1)C(=O)C(=O)N2Cc1cn(CCC(O)COc2cc(=O)oc3ccccc23)nn1
**6c7**	22	0.260	*N*-oxidation	Cc1ccc2c(c1)C(=O)C(=O)N2Cc1cn(CCCCOc2cc(=O)oc3ccccc23)n[n+]1[O-]
**6c7**	23	0.240	alkyl dehydrogenation	Cc1ccc2c(c1)C(=O)C(=O)N2Cc1cn(CCC=COc2cc(=O)oc3ccccc23)nn1
**6c7**	24	0.220	epoxidation	Cc1ccc2c(c1)C(=O)C(=O)N2Cc1cn(CCCCOC23OC2C(=O)Oc2ccccc23)nn1
**6c7**	25	0.210	aliphatic hydroxylation	Cc1ccc2c(c1)C(=O)C(=O)N2Cc1cn(C(O)CCCOc2cc(=O)oc3ccccc23)nn1
**6c7**	26	0.128	dehydration of N-C bond	Cc1ccc2c(c1)C(=O)C(=O)[N+]2=Cc1cn(CCCCOc2cc(=O)oc3ccccc23)nn1
**6c7**	27	0.120	aromatic hydroxylation	Cc1ccc2c(c1)C(=O)C(=O)N2Cc1nnn(CCCCOc2cc(=O)oc3ccccc23)c1O
**6c7**	28	0.084	alkyl dehydrogenation	Cc1ccc2c(c1)C(=O)C(=O)N2Cc1cn(C=CCCOc2cc(=O)oc3ccccc23)nn1
**6c7**	28	0.084	alkyl dehydrogenation	Cc1ccc2c(c1)C(=O)C(=O)N2Cc1cn(CC=CCOc2cc(=O)oc3ccccc23)nn1

For readability, the parent SMILES is reported once at the beginning of each compound block and the predicted metabolite SMILES is listed for each ranked transformation. GLORY output is presented in the rank order generated in MaxCoverage mode. Priority scores are model-derived ranking values and should not be interpreted as experimental probabilities.

(A)

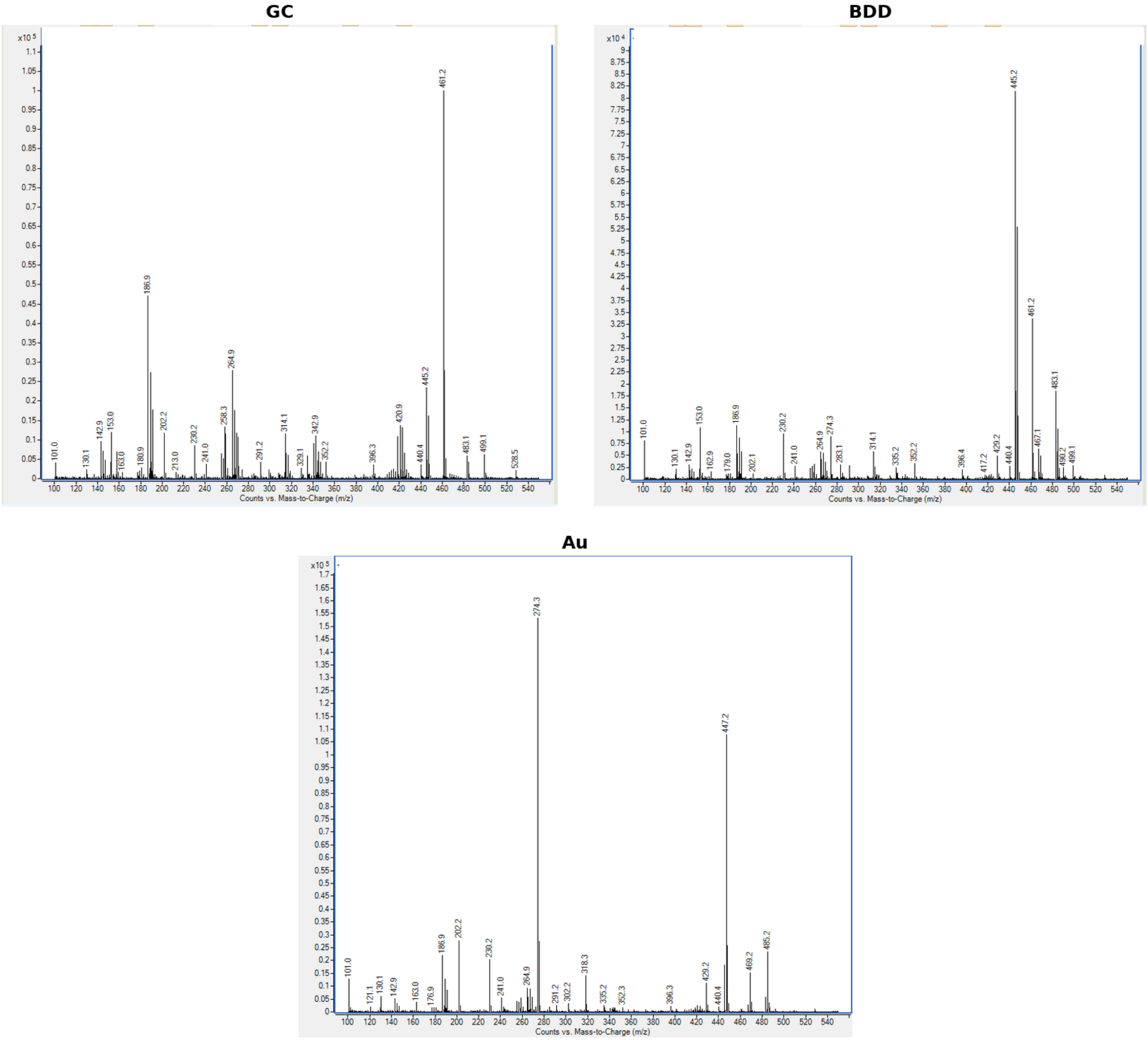
GC
BDD
Au
Counts vs. Mass-to-Charge (m/z)
Counts vs. Mass-to-Charge (m/z)
Counts vs. Mass-to-Charge (m/z)

(B)

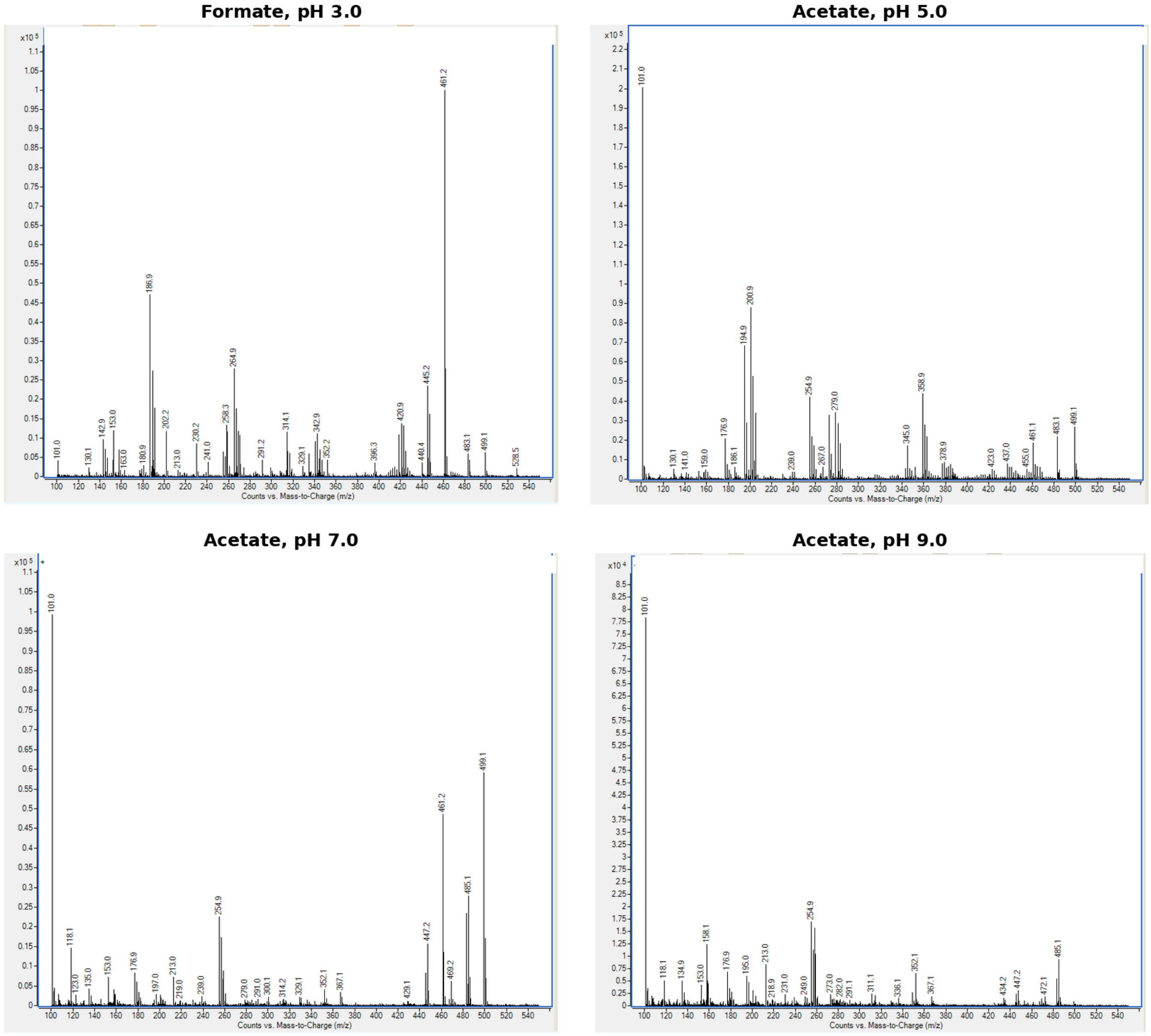

Formate, pH 3.0
101.0
130.1
142.9
153.0
163.0
180.9
186.9
202.2
213.0
230.2
241.0
258.3
264.9
291.2
314.1
329.1
342.9
352.2
396.3
420.9
440.4
445.2
461.2
483.1
499.1
528.5
Counts vs. Mass-to-Charge (m/z)
Acetate, pH 5.0
101.0
130.1
141.0
159.0
176.9
186.1
194.9
200.9
239.0
254.9
267.0
279.0
345.0
358.9
378.9
423.0
437.0
455.0
461.1
483.1
499.1
Counts vs. Mass-to-Charge (m/z)
Acetate, pH 7.0
101.0
118.1
123.0
135.0
153.0
176.9
197.0
213.0
219.0
239.0
254.9
279.0
291.0
300.1
314.2
329.1
352.1
367.1
429.1
447.2
461.2
469.2
485.1
499.1
Counts vs. Mass-to-Charge (m/z)
Acetate, pH 9.0
101.0
118.1
134.9
153.0
158.1
176.9
195.0
213.0
218.9
231.0
249.0
254.9
273.0
282.0
291.1
311.1
336.1
352.1
367.1
434.2
447.2
472.1
485.1
Counts vs. Mass-to-Charge (m/z)

(C)

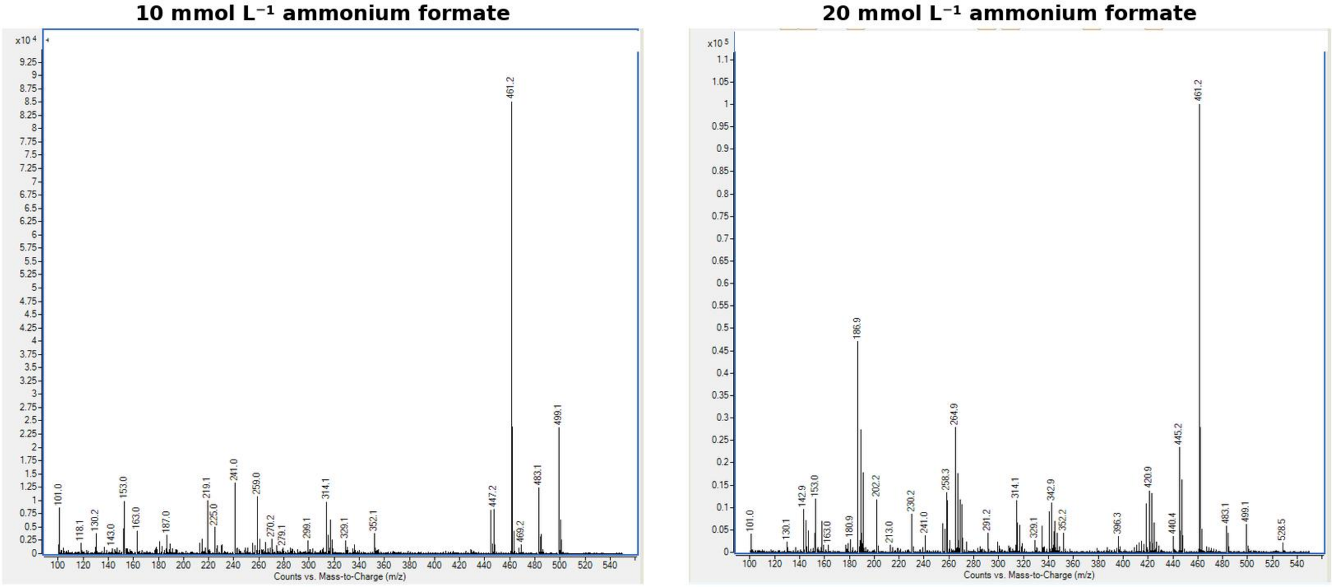

(D)

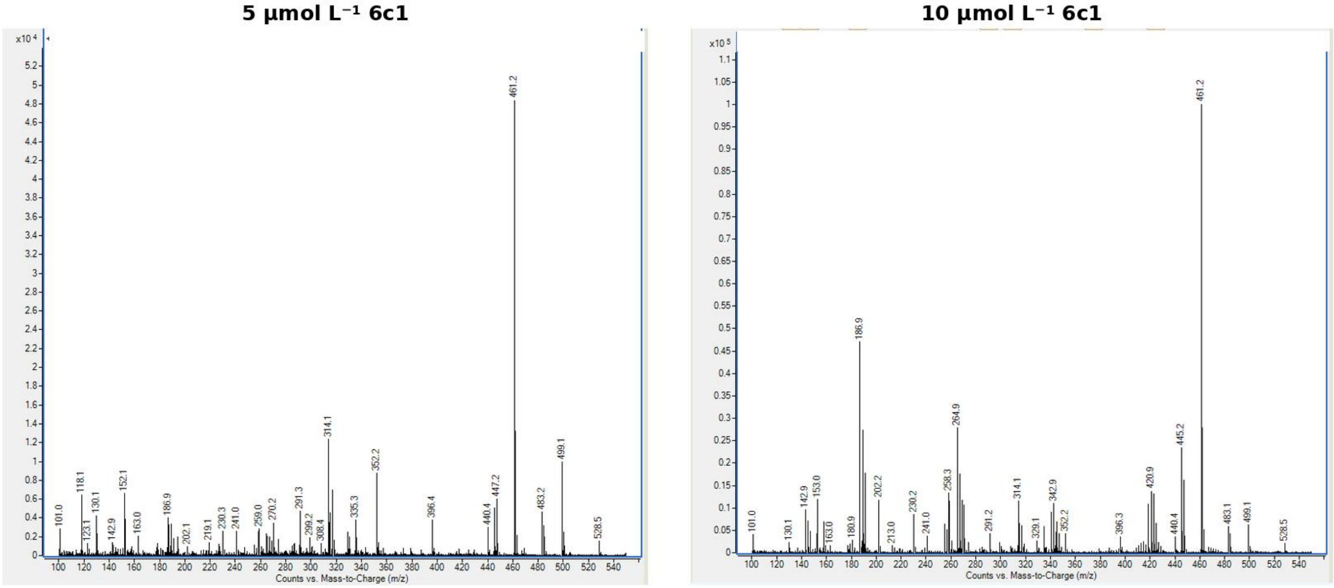

Figure S1. Full-scan positive-ion ESI mass spectra supporting optimization of electrochemical oxidation conditions for compound 6c1. (A) Effect of working-electrode material: glassy carbon (GC), boron-doped diamond (BDD), and gold (Au), using 20 mmol L^{-1} ammonium formate at pH 3.0, 10 µmol L^{-1} 6c1, and 1.7 V vs. HyREF. (B) Effect of reaction medium using the GC electrode at 1.7 V: ammonium formate at pH 3.0 and ammonium acetate at pH 5.0, 7.0, and 9.0. (C) Effect of ammonium formate concentration (10 and 20 mmol L^{-1}) at pH 3.0 using the GC electrode, 10 µmol L^{-1} 6c1, and 1.7 V. (D) Effect of 6c1 concentration (5 and 10 µmol L^{-1}) using the GC electrode, 20 mmol L^{-1} ammonium formate at pH 3.0, and 1.7 V.
